# Peripheral blood CD4^+^CCR6^+^ compartment differentiates HIV-1 infected or seropositive elite controllers from long-term successfully treated individuals

**DOI:** 10.1038/s42003-022-03315-x

**Published:** 2022-04-13

**Authors:** Sara Svensson Akusjärvi, Shuba Krishnan, Bianca B. Jütte, Anoop T. Ambikan, Soham Gupta, Jimmy Esneider Rodriguez, Ákos Végvári, Maike Sperk, Piotr Nowak, Jan Vesterbacka, J. Peter Svensson, Anders Sönnerborg, Ujjwal Neogi

**Affiliations:** 1 Division of Clinical Microbiology, Department of Laboratory Medicine, Karolinska Institutet, ANA Futura, Campus Flemingsberg, 141 52 Stockholm, Sweden; 2 Department of Biosciences and Nutrition, Karolinska Institutet, Neo, Campus Flemingsberg, 141 83 Stockholm, Sweden; 3 Division of Chemistry I, Department of Medical Biochemistry and Biophysics, Karolinska Institutet, Campus Solna, 171 65 Stockholm, Sweden; 4 Division of Infectious Disease, Department of Medicine Huddinge, Karolinska Institutet, I73, Karolinska University Hospital, 141 86 Stockholm, Sweden; 5 Christopher S. Bond Life Sciences Centre, University of Missouri, Columbia, MO 65211 USA; 6 Manipal Institute of Virology (MIV), Manipal Academy of Higher Education, Manipal, Karnataka India

**Keywords:** Infection, HIV infections

## Abstract

HIV-1 infection induces a chronic inflammatory environment not restored by suppressive antiretroviral therapy (ART). As of today, the effect of viral suppression and immune reconstitution in people living with HIV-1 (PLWH) has been well described but not completely understood. Herein, we show how PLWH who naturally control the virus (PLWH_EC_) have a reduced proportion of CD4^+^CCR6^+^ and CD8^+^CCR6^+^ cells compared to PLWH on suppressive ART (PLWH_ART_) and HIV-1 negative controls (HC). Expression of CCR2 was reduced on both CD4^+^, CD8^+^ and classical monocytes in PLWH_EC_ compared to PLWH_ART_ and HC. Longer suppressive therapy, measured in the same patients, decreased number of cells expressing CCR2 on all monocytic cell populations while expression on CD8^+^ T cells increased. Furthermore, the CD4^+^CCR6^+^/CCR6^−^ cells exhibited a unique proteomic profile with a modulated energy metabolism in PLWH_EC_ compared to PLWH_ART_ independent of CCR6 status. The CD4^+^CCR6^+^ cells also showed an enrichment in proteins involved in apoptosis and p53 signalling in PLWH_EC_ compared to PLWH_ART_, indicative of increased sensitivity towards cell death mechanisms. Collectively, this data shows how PLWH_EC_ have a unique chemokine receptor profile that may aid in facilitating natural control of HIV-1 infection.

## Abstract (web summary)

The expression profiles dynamics of several chemokine receptors are lower for people living with HIV-1 who naturally control the virus compared to those on suppressive antiretroviral therapy and HIV-negative controls, shedding light on the mechanisms of natural control of HIV-1 infection.

## Introduction

Efficient antiretroviral therapy (ART) suppresses human immunodeficiency virus type 1 (HIV-1) replication to below the detection level of sensitive HIV RNA assays. Still, HIV-1 persists for decades despite efficient ART in presumably latently infected cells, making up the so-called latent viral reservoir. This reservoir resides in subtypes of memory T cells and cells from monocytic lineages, but also in some functional and naïve T cell subsets^1^. Eradication of latently infected cells, i.e. a sterilizing cure, has proven to be a major hurdle due to several factors such as heterogeneity in mechanisms governing latency and integration sites into the host genome^2^. Over the course of suppressive ART, persisting HIV-1 sustained by low levels of viral replication, homoeostatic proliferation, and cell-to-cell transmission, is a driver of chronic immune activation in the host^3–5^.

In HIV-1 infection, chemokine receptors are essential co-receptors needed for HIV-1 entry into the cell where the CC chemokine receptor 5 (CCR5) and CXC chemokine receptor 4 (CXCR4) are the main co-receptors used for HIV-1 entry, but alternative receptors such as CCR3 may play a role for macrophage-tropic viral strains^6–8^. T cells expressing the chemokine receptor CCR6 are also overrepresented amongst cell types infected by HIV-1^9^. Furthermore, CD4^+^CCR6^+^ and CD4^+^CCR2^+^CCR5^+^ cells have been proposed to contribute to the HIV-1 reservoir^10,11^.

Alterations in chemokine receptor expression and chemokine levels modulate the activity of the immune response and inflammatory levels during HIV-1 infection^12^. In people living with HIV-1 (PLWH) on suppressive ART, these aberrations may contribute to (i) higher risk of age-associated co-morbidities together with elevated immune activation, (ii) microbiome dysbiosis leading to microbial translocation and (iii) dysregulated immune cell activation and function^13–16^. Consequently, HIV-1 pathogenesis and immune dysfunction promote chronic immune activation. This results in a vicious cycle as immune activation likely drives HIV-1 disease progression and inflammaging, defined as an age-related increase of inflammatory markers, that could possibly lead to earlier onset of age-related diseases. In our earlier study on a small group of 10 PLWH on ART for two decades, we reported normalisation of most of the plasma pro-inflammatory cytokines and chemokines to the level of HIV-negative controls^17^. Therefore, although there are several studies on immune reconstitution and viral suppression during short-term ART, the long-term effect of ART on the immune system is not known.

Eradication of latently infected cells, i.e., a sterilising cure, has proven to be a major hurdle. An alternative approach is a functional cure aimed at suppressing viral replication without ART. A model for functional cure studies is elite controllers (herein PLWH_EC_). PLWH_EC_ constitute a small fraction (<0.5%) of HIV-1^+^ individuals able to naturally control viral replication in the absence of ART^18,19^. However, there is a population-based heterogeneity of how these individuals suppress viral replication which is likely caused by several factors such as viral and host genetic factors, variability of integration site in the human genome, and variation in the host response such as immunological factors^20–23^. In terms of immune cell activation, studies have shown how PLWH_EC_ maintain lower levels of inflammatory markers compared to PLWH who progress in their disease^24^. However, as no consensus exists on how the PLWH_EC_ phenotype is maintained, further studies comparing variability of immune function between PLWH_EC_ and PLWH on suppressive ART (herein PLWH_ART_) are required to understand the mechanisms of natural control of HIV-1.

In our recent study^25^, we hypothesised that an altered CCR6/CCL20 chemokine axis and CCR2-CCL7-CCL2 signalling may play a protective role in the PLWH_EC_ phenotype. We identified a downregulation of CCR2 and CCR6 receptors on CD4^+^ and CD8^+^ lymphocytes and higher plasma abundance of CCL4, CCL7 and CCL20 in PLWH_EC_ compared to the HIV-1-negative controls. This can provide natural resistance to HIV-1 infection although the expression profile during long-term suppressive ART is not known. In the present study, we extended our analysis to evaluate the expression profile dynamics of key chemokine receptors CCR2, CCR3, CCR5 and CCR6 in PLWH_ART_. We also compared the proportion of integrated HIV-1 between PLWH_EC_ and PLWH_ART_. Furthermore, cell populations of interest were isolated for quantitative proteomics to evaluate specific characteristics regulating these cell populations and their potential role in HIV-1 persistence. Our study provides important understanding of the chemokine receptor dynamics and its role in HIV-1 persistence that differentiate PLWH_EC_ from PLWH_ART_.

## Results

### PLWH_EC_ have reduced populations of CD4^+^CCR6^+^ and CD4^+^CCR2^+^ T lymphocytes compared to successfully long-term treated patients

**Fig. 1:**
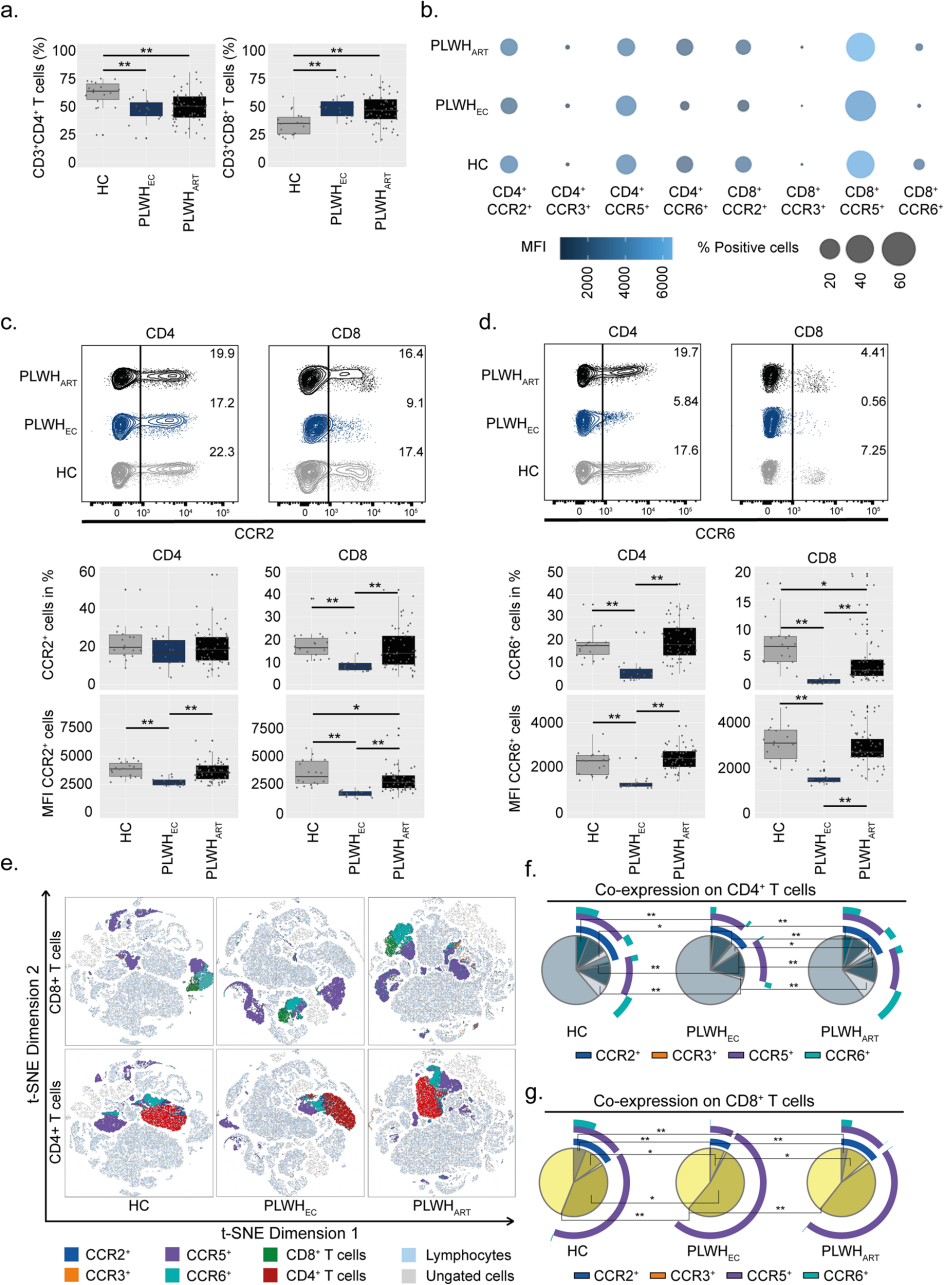
CCR2 and CCR6 are specifically depleted from T lymphocytes in PLWH_EC_. Receptor expression on CD4^+^ and CD8^+^ T lymphocytes from PLWH_EC_ (*n* = 14), PLWH_ART_ (*n* = 54) and HC (*n* = 18). **a** Frequencies of CD4^+^ T cells and CD8^+^ T cells in total peripheral blood mononuclear cells derived from CD3^+^ cells. **b** Bubble chart representing receptor expression of CCR2, CCR3, CCR5 and CCR6 on CD4^+^ and CD8^+^ T cells. Size of the bubble corresponds to percentage-positive cells while colour represents the median fluorescence intensity (MFI). **c** CCR2 receptor expression on CD4^+^ and CD8^+^ T cells (% cells and MFI). **d** CCR6 receptor expression on CD4^+^ and CD8^+^ T cells (% cells and MFI). **e** t-SNE plots showing receptor expression distribution on CD4^+^ and CD8^+^ T cells. **f** Co-receptor expression of CCR2, CCR3, CCR5 and CCR6 on CD4^+^ T cells. **g** Co-receptor expression of CCR2, CCR3, CCR5, and CCR6 on CD8^+^ T cells. **c**, **d** Contour plots show a representing sample from PLWH_EC_, PLWH_ART_ and HC corresponding to the median % of cells within each group. **a**, **c**, **d**, **f**, **g** Statistical significance was determined using two-tailed Mann–Whitney U-test (significance level *p* < 0.05, with *<0.05, **<0.001) and represented as pie charts or with median using 95% CI.

Herein, we wanted to evaluate if there are any differences in the expression profile of some key chemokine receptors between PLWH with natural control of the virus and those on ART. Therefore, we investigated the expression levels of CCR2, CCR3, CCR5 and CCR6 in peripheral blood mononuclear cells (PBMCs) from PLWH_EC_ (*n* = 14), PLWH_ART_ (*n* = 54) and HIV-negative individuals (HC, *n* = 18) by flow cytometry (Supplementary Fig. 1). The clinical and demographic data are presented in Table 1 and Supplementary Table 1. All the PLWH_ART_ were successfully treated at the time of sample collection and plasma viral load was below the detection level (<40 copies/mL). Initial discrimination between CD4^+^ T cells (CD3^+^CD4^+^) and CD8^+^ T cells (CD3^+^CD8^+^) showed that both PLWH_EC_ and PLWH_ART_ exhibited a reduced proportion of CD4^+^ T cells and elevated proportion of CD8^+^ T cells compared to HC (Fig. 1a). Single receptor expression in PLWH_EC_ showed a distinct profile compared to both PLWH_ART_ and HC (Fig. 1b). Expression of CCR2 and CCR6 was reduced on both CD4^+^ and CD8^+^ T cells together with a decreased proportion of CD8^+^CCR2^+^, CD4^+^CCR6^+^ and CD8^+^CCR6^+^ in PLWH_EC_ compared to both PLWH_ART_ and HC (Fig. 1c, d). The expression levels of CCR2 were also reduced on CD8^+^ T cells in PLWH_ART_ compared to HC, as well as the proportion of CD8^+^CCR6^+^ cells (Fig. 1c, d). Even as the total frequency of CD4^+^CCR3^+^ was low in all three groups, the expression of CCR3 was high in both PLWH_EC_ and PLWH_ART_ compared to HC (Supplementary Fig. 2a). Furthermore, expression levels of CCR5 were lower on CD8^+^ T cells in PLWH_EC_ compared to PLWH_ART_ (Supplementary Fig. 2b), while no difference was detected on CD4^+^ T cells. Collectively, these data show how PLWH_EC_ have a unique expression profile of CCR2 and CCR6 while the expression signature of CCR3 is more similar to PLWH_ART_ on lymphocytic cells (Fig. 1e). Continuing, co-expression analysis on CD4^+^ cells showed that CCR2^+^CCR5^+^CCR6^+^, CCR2^+^CCR6^+^ and CCR5^+^CCR6^+^ were reduced in PLWH_EC_ compared to both PLWH_ART_ and HC, while no differences were detected between PLWH_ART_ and HC (Fig. 1f). Furthermore, single receptor expression of CCR2 was reduced in PLWH_EC_ compared to PLWH_ART_ and CCR6 expression was reduced in PLWH_EC_ compared to both PLWH_ART_ and HC (Fig. 1f). Within the CD8^+^ T cell population, co-receptor expression of CCR2^+^CCR5^+^CCR6^+^ was reduced in both PLWH_ART_ and PLWH_EC_ compared to HC while CCR2^+^CCR5^+^ was reduced in PLWH_EC_ compared to HC and PLWH_ART_ (Fig. 1g). Single receptor expression analysis also showed that CCR5 was increased in PLWH_EC_ compared to HC and CCR6 expression decreased in PLWH_EC_ compared to both HC and PLWH_ART_ (Fig. 1g). As the major differences were detected on CCR2 and CCR6 expression, we measured the levels of their ligands, namely monocyte chemoattractant protein 1, MCP1 (CCL2) and macrophage inflammatory protein 3, MIP-3 (CCL20), respectively, in plasma from PLWH_ART_ (*n* = 49), PLWH_EC_ (*n* = 12) and HC (*n* = 16). Plasma levels of CCL20 were increased in both PLWH_EC_ and PLWH_ART_ compared to HC, while no significant difference was detected for CCL2 (Supplementary Fig. 2c, d). In summary, the receptor expression profile on lymphocytes in PLWH_ART_ resembles HC, while PLWH_EC_ exhibits a distinct expression profile with decreased receptor expression of both CCR2 and CCR6.

**Table 1:** Patient characteristics.

Parameter	PLWH_EC_	PLWH_ART_	HC	*P*-values
*N*	14	54	18	ND
Gender: Female, *N* (%)	7 (50)	19 (35)	9 (50)	ND
Ethnicity, *N* (%)				
Black	9 (64.2)	21 (38.8)	5 (27.7)	ND
Asian	–	2 (3.7)	–	
Caucasian	4 (28.6)	31 (57.4)	13 (72.2)	
Hispanic	1 (7.1)	–	–	
*At sampling*				
Age in years, mean (SD)	46 (10.5)	50 (9.5)	47 (9)	0.2704^a^
CD4^+^ T cell count (cells/µL); median (IQR)	680 (570–1060)	660 (485–795)	NA	0.5012^b^
CD8^+^ T cell count (cells/µL); median (IQR)	900 (540–1330)	600 (415–950)	NA	0.0974^b^
CD4:CD8 ratio; median (IQR)	1 (0.44–1.18)	1 (0.7–1.45)	NA	0.3295^b^
Duration of treatment in years; median (IQR)	NA	8 (6.75–19)	NA	ND
Duration of suppressive treatment in years; median (IQR)	NA	8 (7–15)	NA	ND
Years HIV positive; median (IQR)	7 (4.5–11.7)	12.5 (9–20)	NA	0.0012^b^
HIV RNA load; Copies/mL (IQR)	<40 copies/mL	<40 copies/mL	NA	ND
Treatment regimen, *N* (%)				
3rd drug,				
Boosted PI		2 (3.7)		
INSTI		30 (55.5)		
NNRTI	NA	21 (38.8)	NA	NA
1st drug,				
ABC		34 (62.9)		
TAF/TDF		16 (29.6)		
Other		4 (7.4)		
*Initiation of treatment*				
CD4^+^ T cell count at treatment initiation (cells/µL); median (IQR)	NA	351 (167.5–522.5)	NA	ND
Viral Load at treatment initiation; Log_10_ copies/mL (IQR)	NA	4.79 (4.06–5.44)	NA	ND

*ABC* abacavir, *IQR* interquartile range, *N* number, *NA* not applicable, *ND* not done, *PI* protease inhibitor, *INSTI* integrase strand transfer inhibitor, *NNRTI* non-nucleoside reverse transcriptase inhibitor, *SD* standard deviation, *TAF* tenofovir alafenamide, *TDF* tenofovir disoproxil.

^a^One-way ANOVA.

^b^Mann–Whitney test.

### CCR2 expression is reduced in PLWH_EC_ compared to PLWH_ART_ on monocytic cell populations

**Fig. 2:**
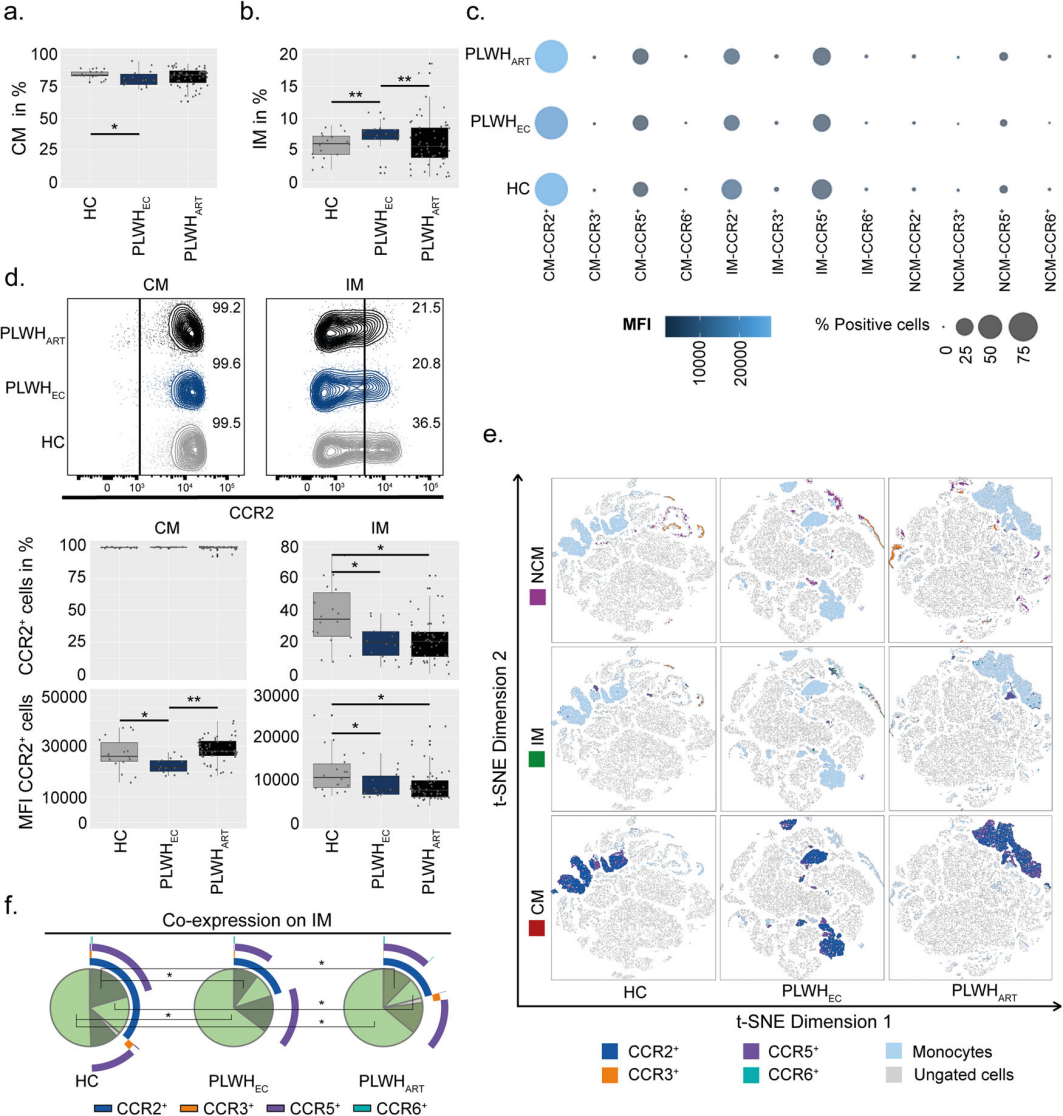
CCR2 is depleted from intermediate monocytes of PLWH. Receptor expression on monocytic subpopulations (classical monocytes (CM); CD14^+^CD16^−^, intermediate monocytes (IM) CD14^+^CD16^+^ and non-classical monocytes (NCM); CD14^−^CD16^+^) in PLWH_EC_ (*n* = 14), PLWH_ART_ (*n* = 54) and HC (*n* = 18). **a**, **b** Percentages of CM (**a**) and IM (**b**) from CD3^−^ cells in peripheral blood mononuclear cells. **c** Bubble chart representing receptor expression of CCR2, CCR3, CCR5 and CCR6 on CM, IM and NCM. Size of the bubble corresponds to percentage-positive cells while colour represents the median fluorescent intensity (MFI). **d** CCR2 receptor expression on CM and IM (% cells and MFI). **e** t-SNE plots showing receptor expression distribution of CCR2, CCR3, CCR5 and CCR6 on CM, IM and NCM. **f** Co-receptor expression of CCR2, CCR3, CCR5 and CCR6 on IM. **d** Contour plot shows a representing sample from PLWH_EC_, PLWH_ART_ and HC corresponding to the median % of cells within each group. **a**, **b**, **d**, **f** Statistical significance was determined using two-tailed Mann–Whitney U-test (significance level *p* < 0.05, with *<0.05, **<0.001) and represented as pie charts or with median using 95% CI.

In HIV-1 infection there is a chronic activation of monocytes contributing to organ-specific inflammatory events that are poorly understood in virologically suppressed patients. Our group and others have previously shown persistent low-level inflammation in PLWH_EC_^24–26^. Furthermore, chemokine receptors like CCR2 and CCR5 play a major role in inflammatory trafficking of monocytes^27^. Therefore, we determined the CCR2, CCR3, CCR5 and CCR6 receptor expression in monocytic cell populations; classical (CM, CD14^+^CD16^−^), intermediate (IM, CD14^+^CD16^+^) and non-classical (NCM, CD14^−^CD16^+^). Herein, PLWH_EC_ exhibited reduced numbers of CM compared to HC and elevated levels of IM compared to both HC and PLWH_ART_ (Fig. 2a, b and Supplementary Fig. 3a). Generally, the receptor expression of CCR3 and CCR6 was low on all three monocytic cell subsets (Fig. 2c). The major difference was a reduced expression of CCR2 on CM and IM in PLWH_EC_ compared to HC and on CM compared to PLWH_ART_ (Fig. 2d). In PLWH_ART_, both the frequency of IM-CCR2^+^ cells and expression of CCR2 on IM cells were reduced compared to HC (Fig. 2d). Although the proportion of CM-CCR6^+^ cells was decreased in PLWH_EC_ compared to PLWH_ART_, CCR6 expression was increased in PLWH_EC_ compared to HC and PLWH_ART_, while PLWH_ART_ showed reduced expression compared to HC (Supplementary Fig. 3b). Furthermore, NCM-CCR6^+^ cells were lower in PLWH_EC_ compared to PLWH_ART_ and HC, and the frequency of CM-CCR3^+^ cells was decreased in PLWH_EC_ compared to PLWH_ART_ (Supplementary Fig. 3b, c). The receptor expression of CCR5 was reduced on IM in PLWH_ART_ compared to HC and on NCM in PLWH_EC_ compared to both PLWH_ART_ and HC (Supplementary Fig. 3d). This data indicates that the main difference in the monocytic cell subsets is a reduced expression of CCR2 in PLWH_EC_ compared to the other groups (Fig. 2e). The IM were the only monocytic cell population which exhibited differences in co-receptor expression. For these cells, the largest variation was a reduction of CCR2^+^CCR5^+^ expressing cells in both PLWH_EC_ and PLWH_ART_ compared to HC. An increase in cells lacking all four receptors was found in both PLWH_EC_ and PLWH_ART_ compared to HC (Fig. 2f). Furthermore, PLWH_ART_ exhibited reduced numbers of cells expressing only CCR2 compared to HC (Fig. 2f). Collectively, this data shows that the largest variation occurred on IM where PLWH_ART_ and PLWH_EC_ showed a similar trend compared to HC.

### Longer suppressive therapy reduces expression of CCR2 on monocytes

**Fig. 3:**
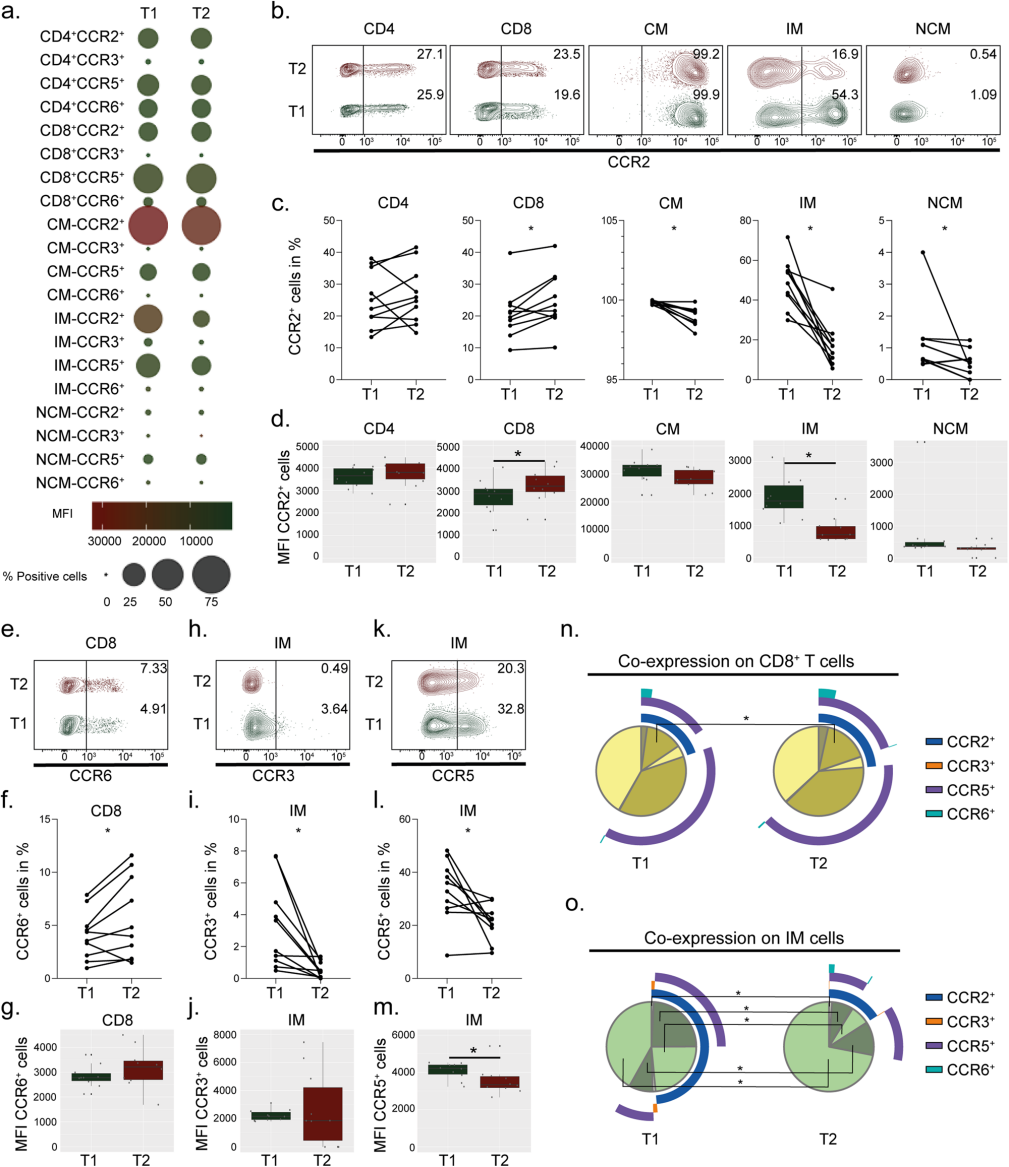
In time, CCR2 and CCR6 increase on CD8 cells while CCR2 decreases on monocytes in PLWH_ART_. Receptor expression of longitudinal data on lymphocytes and monocytes at arbitrary timepoint 1 (T1) (*n* = 10) and three years later at timepoint 2 (T2) (*n* = 10) from total peripheral blood mononuclear cells. **a** Bubble chart representing receptor expression of CCR2, CCR3, CCR5 and CCR6 on CD4^+^ and CD8^+^ T cells together with classical (CM; CD14^+^CD16^−^), intermediate (IM; CD14^+^CD16^+^), and non-classical (NCM; CD14^−^CD16^+^) monocytes. Size of the bubble corresponds to percentage-positive cells while colour represents the median fluorescent intensity (MFI). **b**, **c** Receptor expression of CCR2 on CD4^+^, CD8^+^, CM, IM and NCM. **d** MFI of CCR2 on CD4^+^, CD8^+^, CM, IM and NCM. **e**, **f** Receptor expression of CCR6 on CD8^+^ T cells. **g** MFI of CCR6 on CD8^+^ T cells. **h**, **i** Receptor expression of CCR3 on IM. **j** MFI of CCR3 on IM. **k**, **l** Receptor expression of CCR5 on IM. **m** MFI of CCR5 on IM. **n**, **o** Co-receptor expression of CCR2, CCR3, CCR5 and CCR6 on CD8^+^ T cells (**n**) and IM (**o**). **b**, **e**, **h**, **k** Contour plot shows a representing sample from T1 and T2 corresponding to the median % of cells within each group. **c**, **d**, **f**, **g**, **i**, **j**, **l**–**o** Statistical significance was determined using two-tailed Wilcoxon matched-pairs signed-rank test (significance level *p* < 0.05, with *<0.05, **<0.001) and represented as paired before and after plot, median using 95% CI or as pie charts.

The chronic inflammatory condition due to HIV-1 persistence and an activated immune response persists during suppressive ART. Therefore, to evaluate if longer treatment duration affects the receptor expression, we included longitudinal samples (timepoint 1 (T1) *n* = 10 and timepoint 2 (T2) *n* = 10) where the same patients were followed-up after 3 years of additional suppressive therapy (clinical parameters can be viewed in Supplementary Table 2). Treatment duration did not affect frequencies of CD4^+^, CD8^+^ T cells or monocytic cell populations (Supplementary Fig. 4a, b). For the detected receptor expressions, most of the differences were detected in the monocytic cell populations while no significant variations were seen on CD4^+^ T cells (Fig. 3a). Longer treatment duration increased the frequency of CD8^+^CCR2^+^ cells, while all three monocytic cell populations exhibited reduced proportions of cells expressing CCR2 in T2 (Fig. 3b, c). Following a similar trend, CCR2 expression was increased on CD8^+^ T cells and IM exhibited reduced expression of CCR2 in T2 while CM and NCM showed no difference (Fig. 3d). The proportion of CD8^+^CCR6^+^ cells was increased in T2, without showing any significant effect on CCR6 expression levels (Fig. 3e–g). Furthermore, the proportions of IM-CCR3^+^ and IM-CCR5^+^ cells decreased over time while only expression levels of CCR5 decreased in T2 (Fig. 3h–m). Investigations of co-receptor expression on the lymphocytic cell populations showed that the only difference was an increase in CCR2^+^CCR5^+^ in T2 on CD8^+^ T cells (Fig. 3n). Within the monocytic cell populations, the only differences of co-expression of the receptors were seen on IM where T2 exhibited higher percentage of cells negative for all four receptors while CCR2^+^, CCR2^+^CCR3^+^ and CCR2^+^CCR5^+^ were decreased in T2 (Fig. 3o). Conclusively, the effect induced by longer suppressive treatment mainly reduces expression levels of these chemokine receptors on IM.

### The HIV-1 reservoir is lower in PLWH_EC_ compared to PLWH_ART_

**Fig. 4:**
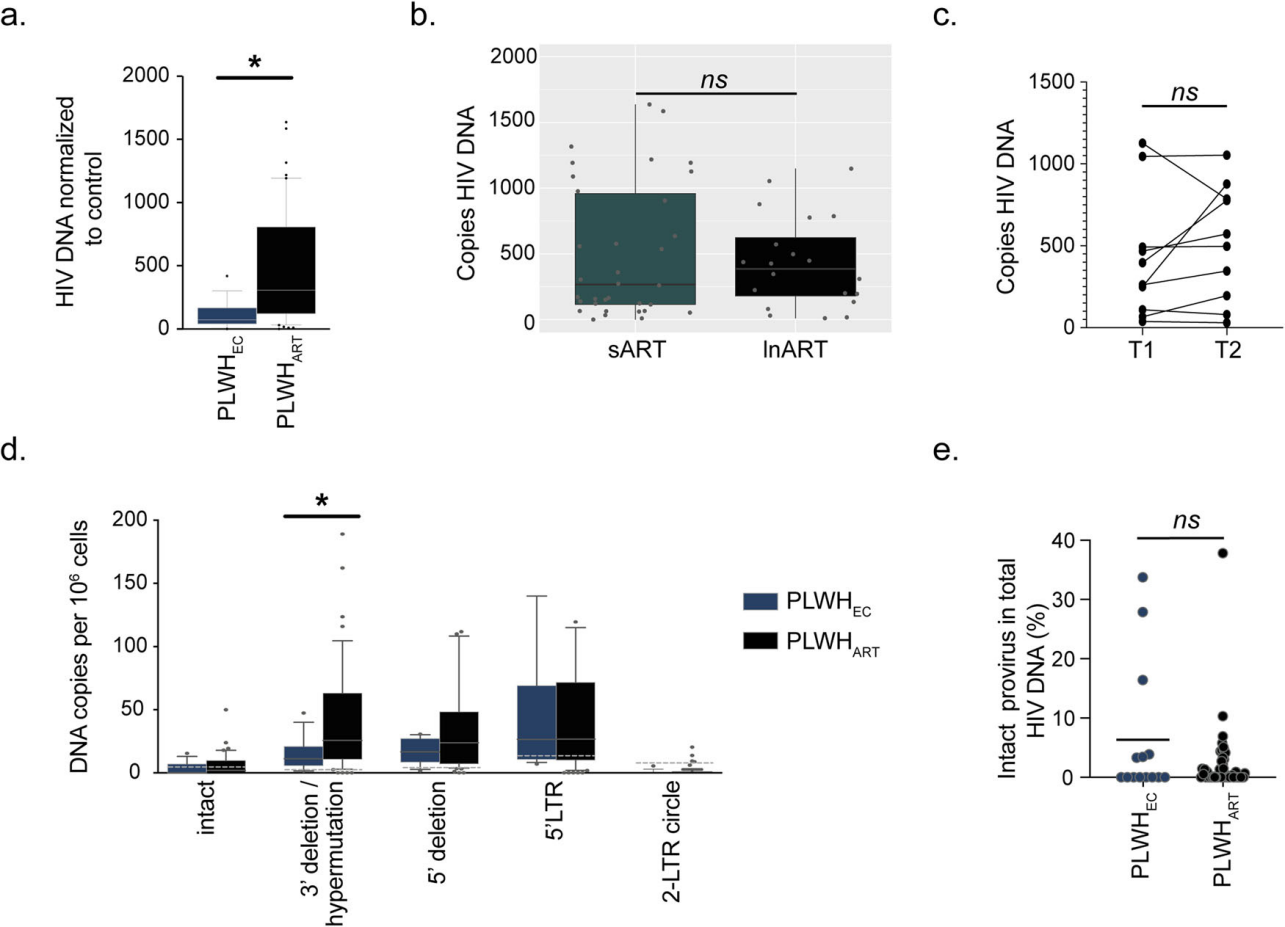
The HIV-1 reservoir in PLWH_EC_ is distinct from PLWH_ART_. **a** Quantified total HIV-1 DNA in peripheral blood mononuclear cells from PLWH_EC_ (*n* = 14) and PLWH_ART_ (*n* = 54). **b** Total HIV-1 DNA in short-term ART (sART, <10 yrs, *n* = 34) compared to long-term ART (lnART, >10 yrs, *n* = 20) in people living with HIV-1 on suppressive therapy. **c** Total HIV-1 DNA in longitudinal data from HIV-1 infected individuals (timepoint 1 (T1), *n* = 10 and timepoint 2 (T2), *n* = 10). **d** Box and Whisker plot show the distribution of integrated HIV-1 DNA copies per 10^6^ PBMCs in PLWH_ART_ (*n* = 54) and PLWH_EC_ (*n* = 14) with whiskers representing the 10–90 percentile. The median is represented by the line inside the box. The dotted line marks the background represented by HC samples. **e** Percentage of intact provirus out of total HIV-1 DNA detected. Statistical significance was determined using two-tailed Mann–Whitney U-test (significance level *p* < 0.05, with *<0.05, **<0.001) and represented as median using 95% CI. All individual samples were run in technical duplicates.

To evaluate the proportion of HIV-1 infected cells in our cohort, we quantified total HIV-1 DNA in the PLWH_EC_ (*n* = 14) and PLWH_ART_ (*n* = 54). Herein, we observed a higher level of total HIV-1 DNA in PLWH_ART_ compared to PLWH_EC_ (Fig. 4a) despite a median of eight years of successful treatment. No significant difference was observed between long-term ART (lnART; >10 yrs (median 20 yrs, Interquartile range (IQR); 18–22.75), *n* = 20) and short-term ART (sART; <10 yrs (median 7 yrs, IQR; 6–8), *n* = 34) nor was the reservoir affected by longer suppressive treatment between T1 and T2 within the same individuals (Fig. 4b, c). This data indicates that after the initial two phases of HIV DNA decline the size of the reservoir is not affected during successful suppressive ART. However, PLWH_EC_ exhibited reduced proportions of HIV-1 infected cells presumably due to their natural control of HIV-1. Next, to evaluate the proportion of integrated HIV-1 that was intact/defective, we employed the intact provirus DNA assay (IPDA)^28^ together with quantification of the 5′LTR and 2-LTR circles on the genomic DNA from PBMCs. A significant increase of 3′deletion/hypermutation was detected in PLWH_ART_ compared to PLWH_EC_, but no significant differences in intact provirus, 5′deletion, 5′LTR or 2-LTR circles between the two groups (Fig. 4d). Moreover, percentages of intact provirus out of total HIV-1 DNA did not differ between the groups (Fig. 4e). The CCL20 ligand for CCR6 can facilitate nuclear integration of HIV-1 by remodelling the actin cytoskeleton, but also directly inhibits infection in the female reproductive tract^29,30^. Therefore, to evaluate if the ligands for CCR2 and CCR6 might have an effect on the HIV-1 reservoir we performed correlation analysis between plasma levels of CCL20 CCL2 and the HIV-1 reservoir. No significant correlation between HIV-1 DNA and CCL20 plasma levels was revealed in either PLWH_ART_ or PLWH_EC_ (Supplementary Fig. 5a). For CCL2, a negative correlation was detected in PLWH_EC_ but not in PLWH_ART_ (Supplementary Fig. 5b). Collectively, this data indicates that although PLWH_ART_ have higher proportion of infected cells compared to PLWH_EC_, a larger fraction of the integrated HIV-1 carries hypermutations or 3′deletions within the viral genome. Furthermore, the frequency of intact provirus out of total HIV-1 DNA is low in both PLWH_EC_ and PLWH_ART_.

### Altered metabolic pathways in CD4^+^CCR6^+^ cells are characterised by an upregulation in p53 signalling and apoptosis in PLWH_EC_

**Fig. 5:**
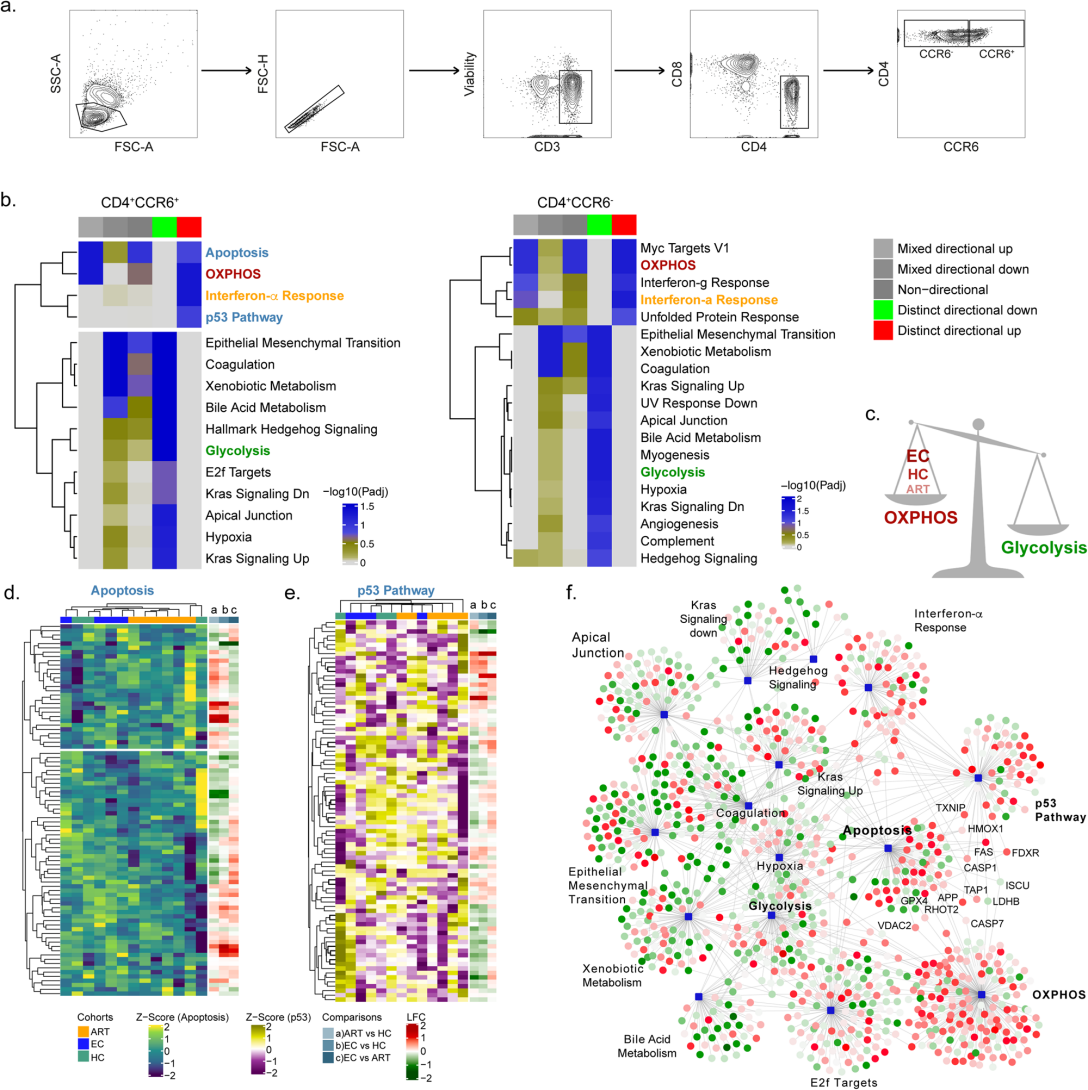
CD4^+^CCR6^+^ are enriched in proteins from p53 and apoptosis signalling in PLWH_EC_ compared to PLWH_ART_. Characteristics of CD4^+^CCR6^+^ and CD4^+^CCR6^−^ cell populations isolated by FACS from PLWH_EC_ (*n* = 4), PLWH_ART_ (*n* = 6) and HC (*n* = 3). **a** Gating strategy for isolation of CD4^+^CCR6^+^ and CD4^+^CCR6^−^ cell populations from total peripheral blood mononuclear cells, exhibited by an HC sample. **b** Heatmap representation of significant pathways found deregulated in CD4^+^CCR6^+^ PLWH_EC_ compared to CD4^+^CCR6^+^ PLWH_ART_ and in CD4^+^CCR6^−^ PLWH_EC_ compared to CD4^+^CCR6^−^ PLWH_ART_ using MSigDBv. Colour gradient corresponds to the negative log scaled adjusted p-values. Each column represents *p*-values of various directionality classes, calculated for the pathways. Non-directional p-values are calculated based on gene-level statistics regardless of the direction of expression. Mixed directional up and mixed directional down p-values are calculated using the subset of the gene statistics that are upregulated and downregulated, respectively. Distinct directional up and distinct directional down p-values are calculated from gene statistics with expression direction. **c** Schematic representation of metabolic trade-off between OXPHOS and glycolysis detected in the cells. **d**, **e** Heatmap representation of proteins part of apoptosis (**d**) and p53 pathway (**e**). Colour gradient corresponds to the z-score scaled normalized expression values. Column annotation represents the cohorts and row annotation visualizes the log2 transformed fold change values in each of the differential expression analysis of PLWH_ART_ compared with HC, PLWH_EC_ compared with HC and PLWH_EC_ compared with PLWH_ART_. **f** Network visualization of significant pathways enriched in CD4^+^CCR6^+^ PLWH_EC_ compared to CD4^+^CCR6^+^ PLWH_ART_. Each edge represents association of the protein with the corresponding pathways. Circular nodes denote proteins and square-shaped nodes are pathways. Red colour gradient and green colour gradient represent upregulation and downregulation of the proteins in CD4^+^CCR6^+^ PLWH_EC_ compared to CD4^+^CCR6^+^ PLWH_ART_.

The subpopulation of CD4^+^CCR6^+^ cells has been described as more permissive towards HIV-1 and targets for productive infection^31,32^. To further characterise the CD4^+^ T cells that express or lack the CCR6 receptor we sorted out these cell populations using fluorescence-activated cell sorting (FACS) from HC (*n* = 3), PLWH_EC_ (*n* = 6) and PLWH_ART_ (*n* = 6) (Fig. 5a). The PLWH_ART_ samples were selected from PLWH on long-term suppressive ART (median 20 years, IQR; 17.5–22.25). First, as the HIV-1 reservoir has been proposed to reside in CD4^+^CCR6^+^ T cells, we measured the amount of integrated HIV-1 in PLWH_EC_ (*n* = 5) and PLWH_ART_ (*n* = 3). However, the variation within the data and the small sample size made the comparison inconclusive (Supplementary Fig. 6a). To evaluate the specific proteomic profile of CD4^+^CCR6^−^ and CD4^+^CCR6^+^ cell populations we isolated these cell populations by FACS (15,000–30,000 cells) and performed mass spectrometry-based proteomics using a quantitative tandem mass tags (TMT) labelling approach. Due to low protein detection by mass spectrometry four PLWH_EC_ samples, two samples out of each of the CCR6^+^ and CCR6^−^ cell populations were excluded from the analysis. The principal component analysis (PCA) identified heterogeneity among the samples (Supplementary Fig. 6b). Differential expression analysis was unable to find statistically significant regulated proteins due to heterogeneity among samples and low sample size. Therefore, functional analysis was performed using a consensus scoring approach based on multiple protein set analysis (PSA) run by incorporating the directionality of protein abundance. Using the group-specific consensus scores (PLWH_EC_ vs PLWH_ART_) and directionality classes, we identified distinct upregulation of oxidative phosphorylation (OXPHOS) (adj *p* < 0.05) and interferon-α response (adj *p* < 0.05) and distinct downregulation of glycolysis (adj *p* < 0.05) in both CD4^+^CCR6^+^ and CD4^+^CCR6^−^ cells while using the MSigDB hallmark gene set (Fig. 5b). Similarly, the KEGG gene set also identified a distinct upregulation of OXPHOS (adj *p* < 0.1), and a distinct downregulation of other pathways of amino acid and carbohydrate metabolism (Supplementary Fig. 6c). This data indicates that the metabolic trade-off of increased OXPHOS and reduced glycolysis is elevated in PLWH_EC_ compared to PLWH_ART_ (Fig. 5c). The relative abundance of the OXPHOS proteins was higher in PLWH_EC_ compared to HC while lower in PLWH_ART_ compared to HC (Supplementary Fig. 7a, b). Interestingly, only p53 signalling and apoptosis were distinctly upregulated in CD4^+^CCR6^+^ (adj *p* < 0.05) (Fig. 5b) and both pathways had unique features in PLWH_EC_ (Fig. 5d, e). The majority of the PLWH_ART_ clustered together. There were two downregulated proteins in PLWH_EC_ (ISCU and LDHB) that were common between OXPHOS and p53 signalling. Furthermore, four proteins (CASP7, VDAC2, RHOT2 and GPX4) were common between OXPHOS and apoptosis, while seven proteins (FAS, TAP1, FDXR, TXNIP, CASP1, APP and HMOX1) were common between p53 signalling and apoptosis among the proteins upregulated in PLWH_EC_ compared to PLWH_ART_ (Fig. 5f). Taken together, this data shows how the CD4^+^CCR6^+^ cell population have a unique profile in PLWH_EC_. The co-regulation of OXPHOS, p53 signalling and apoptosis may be a contributing factor to control HIV-1 infection.

## Discussion

In our study, a unique cell type expression profile of CCR2 and CCR6 was identified in PLWH_EC_, while PLWH_ART_ exhibited an expression profile more like HC. CCR6^+^ cells were thus decreased on both CD4^+^ and CD8^+^ T cells in PLWH_EC_ but not in PLWH_ART_. Furthermore, the CD4^+^ T cells exhibited a protein profile of modulated metabolic activity towards OXPHOS, and reduced protein levels involved in glycolysis in PLWH_EC_, irrespective of CCR6 status. Collectively, as CD4^+^CCR6^+^ cells have been reported to be highly permissive to HIV-1^31,32^, and substantial contributors to the HIV-1 reservoir, low levels of CD4^+^CCR6^+^ cells, metabolic modulation, and enrichment of proteins involved in cell death, could potentially contribute to natural control of HIV-1.

Within the CD4^+^ T cell subpopulation, CCR6 expression is a marker of some memory T cells subsets, namely Th17 cells, natural killer T (NKT) cells, and γδ T cells^33^. Of these cell populations, the Th17 cells promote a strong pro-inflammatory response and are drivers of chronic inflammation and autoimmune diseases^34,35^. Earlier studies have shown how memory CD4^+^CCR6^+^ T cells express a Th17 lineage profile and high susceptibility towards HIV-1^36^. In our cohort, the proportion of CCR6^+^ cells was reduced in PLWH_EC_ compared to both PLWH_ART_ and HC. CCR6 is also a homing marker for gut-associated lymphoid tissue (GALT)^37^. Therefore, the causative factor of reduced CD4^+^CCR6^+^ cells in peripheral blood in PLWH_EC_ could be tissue residency in GALT to a higher extent than in PLWH_ART_. Although initiation of ART restores the intestinal CD4^+^ T cell compartments, the severe depletion induced during the early stages of infection cannot be fully recovered^38^, which contributes to exacerbation of HIV-1 through microbial translocation and hyperactivation of the immune system^39–41^. Collectively, an increased gut homing potential of CCR6^+^ cells in PLWH_EC_, could help to restore the imbalance of CD4^+^ T cells in GALT and reduce systemic chronic inflammation among these patients.

We have earlier shown that low expression of CCR6 and high levels of its ligand, CCL20, may result in low levels of inflammation in PLWH_EC_^25^. Herein, we detected increased plasma levels of CCL20 in PLWH, independent of HIV-1 status compared to HC, confirming earlier observations^42^. CCL20 has previously been described to have an effect on the infection steps of HIV-1 and migration/function of immune cells^33^. However, we did not see any difference in CCL20 levels between PLWH_EC_ and PLWH_ART_. Therefore, most likely it is the CD4^+^CCR6^+^ cells, rather than levels of CCL20, that in PLWH_EC_ could contribute to natural control of HIV-1.

Furthermore, CD4^+^CCR6^+^ and CD8^+^CCR6^+^ cells decline in untreated patients with HIV-1^43^ since the CCR6^+^ cells are more susceptible towards apoptosis and therefore involved in the depletion of memory T cells. However, we did not detect any alterations in CD4^+^CCR6^+^ compartments in PLWH_ART_ compared to HC, although for CD8^+^CCR6^+^ cells there was a reduction in both patient groups. We identified the CD4^+^CCR6^+^ cells as having increased proteins involved in apoptosis and p53 signalling in PLWH_EC_, indicating an increased sensitivity towards apoptosis, possibly as a result of HIV-1 proteins, activation-induced cell death or induced expression of death receptors, e.g., Fas and TRAIL-RI/TRAIL-RII^44^. Since these cells are not restored in the GALT during ART, it can be postulated that the CD4^+^CCR6^+^ cell population is a major target for HIV-1 infection that in PLWH contributes to the inflammatory environment in both GALT and peripheral blood. In PLWH_EC_, these cells may be more susceptible towards apoptosis, possibly through expression of pro-apoptotic HIV-1 proteins during infection or reactivation from latency. Furthermore, p53 is a tumour suppressor gene that during HIV-1 infection is activated by interferon α and β stimulation of immune cells^45^. Activity of p53 can interfere with HIV-1 replication by inhibiting reverse transcription of the virus and suppressing the activity of the transactivator tat, needed for viral transcription^46^. Therefore, an enrichment of p53 signalling in PLWH_EC_ could contribute to natural control of HIV-1 infection in CD4^+^CCR6^+^ T cells by reducing replication of HIV-1 and inducing apoptosis upon latency reversal. However, the sample size included in this study is relatively small and further studies validating these results are warranted. In addition, PLWH_EC_ exhibited an enrichment of proteins involved in OXPHOS together with decreased number of proteins involved in glycolysis, irrespective of CCR6 expression. Increased glycolysis is a hallmark for T cell activation but also upregulated during HIV-1 infection for cells to sustain the energy-demanding process of virus production^47,48^. Generally, activation of a T cell induces a shift from OXPHOS to aerobic glycolysis in a similar fashion as the metabolic reprogramming occurring during activation from latency^49,50^. Therefore, the metabolic state of the CD4^+^ T cell compartment in PLWH_EC_, independently of CCR6 expression indicates a unique proportion of CD4 T cell subsets and metabolic reprogramming.

Persistent HIV-1 is a driver of chronic immune activation and inflammation. In our cohort, PLWH_EC_ had a reduced amount of integrated HIV-1 in total PBMCs compared to PLWH_ART_, correlating to earlier observations^23^. On the other hand, treatment duration did not significantly alter the proportion of latently infected cells probably due to the stability of the reservoir during long-term ART. During suppressive therapy, persisting HIV-1 contributes to low levels of viral replication and cell-to-cell spread driving the chronic immune cell activation and inflammaging that ultimately sustains the HIV-1 reservoir in the body, together with homoeostatic proliferation^51,52^. However, evaluation of the intact provirus by IPDA showed a decrease of 3′deletion/hypermutation in the provirus from PLWH_EC_ compared to PLWH_ART_. Earlier studies have shown that 3′ deletion clones decline during suppressive therapy^53^. These specific mutated proviruses may be under negative selection due to their capacity to stimulate the immune system^53,54^. With a reduced proportion of provirus in PLWH_EC_ compared to PLWH_ART_, it could be hypothesised that this negative selection is stronger in PLWH_EC_, explaining the lower proportion of integrated HIV-1.

Monocytes produce pro-inflammatory cytokines where IM are efficient producers of IL6 and IL8 in response to microbial pathogens together with CM, whereas NCM produce TNFα, CCL3 and IL-1β in response to viral elements^55^. In our cohort, elevated levels of IM were detected in PLWH_EC_, indicative of an increased pro-inflammatory response. However, the expression profile of CCR2 on IM was similar between PLWH_EC_ and PLWH_ART_. Furthermore, expression of CCR2 decreased on IM during longer suppressive therapy in our longitudinal PLWH_ART_ cohort and a similar trend was seen in PLWH_EC_ and PLWH_ART_ compared to HC. Monocytes are targets of HIV-1 infection and drivers of inflammation and comorbidities during HIV-1 infection^56^. Therefore, the expansion of IM could indicate a role in a heightened pathogen defence in PLWH_EC_.

Overall, our study showed that the chemokine receptor profile in PLWH_ART_ is more similar towards HC on lymphocytic cell populations, and PLWH_EC_ on monocytic cell populations. PLWH_EC_ have a reduced proportion of CD4^+^CCR6^+^ cells in peripheral blood compared to PLWH_ART_. Based on our proof of principle proteomic analysis we hypothesise that the CD4^+^CCR6^+^ cells are more susceptible to apoptosis, while CD4^+^ T cells in PLWH_EC_ (CCR6^−^/CCR6^+^) exhibit decreased metabolic activation, indicative of a reduced activation state. Collectively, these lymphocytic cell populations may aid in facilitating the unique characteristics of PLWH_EC_ and contribute to chronic inflammation and HIV-1 persistence during suppressive therapy.

## Methods

### Patient material

The clinical cohort consisted of HIV-1^+^ individuals on suppressive therapy (PLWH_ART_, *n* = 54), elite controllers (PLWH_EC_, *n* = 14) collected from the InfCareHIV cohort at the Department of Infectious Diseases, Karolinska University Hospital, Sweden, and HIV-1 negative individuals (HC, *n* = 18). The cohort was age-matched and the median duration of treatment was 8 years (IQR; 6.75–19). Cohort characteristics can be viewed in Table 1 and treatment regimen for PLWH_ART_ in Supplementary Table 1. The cohort also consisted of longitudinal data within the PLWH_ART_ group (T1, *n* = 10 and T2, *n* = 10) (Supplementary Table 2). At the time of sampling, none of the study participants had any co-infections. Patient material consisted of plasma and PBMCs collected from whole blood using Ficoll-Paque (Cytiva).

This study was approved by the Regional Ethics Committee of Stockholm. Informed consent was given from all participants prior to inclusion, and data were anonymized and delinked before analysis.

### Flow cytometry and FACS

PBMCs were stained for cell surface markers for 20 min at room temperature (RT) using 5 µL/10^6^ PBMCs of CD3 (OKT3, FITC, Biolegend #317306), CD4 (SK3, BUV395, BDbioscience #563550), CD8 (RPA-T8, APC, Biolegend #301014), CD14 (M5E2, BV510, Biolegend #301842), CD16 (3G8, BV786, BDbioscience #563690), CCR2 (1D9, BB700, BDbioscience #747847), CCR3 (5E8, BV421, Biolegend #310714), CCR5 (2D7, PE-CF594, BDbioscience #562456), CCR6 (G034E3, BV711, Biolegend #353436), and Near IR viability dye (Invitrogen #L10119). All antibodies were used at a volume of 5 µL/100 µL reaction and Near IR viability dye at a concentration of 1:100. Cells were subsequently washed two times using FACS buffer (PBS with 2% FBS and 2 mM EDTA) and fixed 15 min at RT using 2% paraformaldehyde. Results were acquired on BD FACS Symphony (BD Bioscience, USA) using laser and filter settings as indicated for BUV395, BV421, BV510, BV711, BV786, FITC, BB700, PE-CF594, APC and Near IR, respectively; 355 nm UV (100 mW) 379/28, 405 nm violet (100 mW), 450/50, 525/50 (505 LP), 710/50 (685 LP), 810/40 (770 LP), 488 nm blue (200 mW) 530/30 (505 LP), 710/50 (685 LP), 561 nm Y/G (200 mW), 610/20 (600LP) and 637 nm Red (140 mW), 670/30 and 780/60 (750LP). Cell expression analysis and t-SNE were performed using FlowJo 10.6 (Tree Star Inc). Co-expression analysis was performed using the Boolean gating function and the visualisation of complex data using Spice^57^. Data from flow cytometry detection in PLWH_EC_ (*n* = 14) and HC (*n* = 8) samples were used from our earlier publication^25^.

Cryopreserved PBMCs were used for fluorescent activated cell sorting (FACS) for subsequent proteomic analysis and DNA extraction. Cells were thawed and stained using anti-human CD3 (OKT3, FITC, Biolegend #317306), CD4 (SK3, PE-Cy5, Biolegend # 344654), CD8 (RPA-T8, BV570, Biolegend #301038), CD14 (M5E2, BV510, Biolegend #301842), CD16 (3G8, BV786, BDbioscience #563690), CCR6 (G034E3, BV711, Biolegend #353436) for 30 min at 4 °C and subsequent FVS450 viability dye (BDbioscience #562247) for 10 min at RT. All antibodies were used at a volume of 5 µL/100 µL reaction and FVS450 was used at 1:1000. CD4^+^CCR6^−^ and CD4^+^CCR6^+^ cell was collected for downstream analysis. Cells were sorted using SONY MA900 (SONY cooperation, Tokyo, Japan).

### DNA extraction

Total DNA was extracted from whole PBMCs or collected cell populations by QIAamp Mini or QIAamp Micro DNA isolation kit (Qiagen), according to the manufacturers protocol.

### HIV-1 DNA quantification

Patient PBMCs or sorted cells were used for quantification of total HIV-1 DNA by Internally Controlled qPCR (IC-qPCR) developed by Vicenti et al.^58^. In brief, total HIV-1 DNA was quantified using Takara Universal mastermix on the ABI 7500F and normalized to β-Actin levels, primer and probe details are described in Supplementary Table 3. For each sample, 200–500 ng were run per reaction in duplicates and data were normalised to background detection in HC.

### Digital droplet PCR and intact proviral DNA assay (IPDA)

Digital droplet PCR (ddPCR) was performed using the QX200 Droplet Digital qPCR System (RioRad). Samples consisting of 10 µL Supermix for probes (no dUTPs) (Bio-Rad), 900 nM primers, 250 nM probe (labelled with either HEX or FAM) or 500 ng of genomic sample DNA and 50 ng of DNA for RPP30 quantification for shearing analysis and normalization, were emulsified with the QX200 droplet generator. PCR reactions were subsequently performed on a C1000 thermal cycler (Bio-Rad) using the following thermal cycling protocol: 10 min at 95 °C for enzyme activation followed by 45 annealing/extension cycles of 30 s at 94 °C and 60 s at 57 °C and a final enzyme deactivation step at 98 °C for 10 min. Subsequently, samples were read with the QX200 Droplet Reader (Bio-Rad) and analysed with the QuantaSoft software version 1.5 (Bio-Rad).

For the quantification of intact, 5′ deleted, and 3′ deleted/hypermutated proviruses, the Intact Proviral DNA Assay was performed as previously described^28^. Briefly, a first ddPCR was performed as described above with primer/probe combinations for the packaging signal (Ψ), which frequently shows small mutations. The second reaction targets the HIV-1 Rev response element, present in the Env coding region. Two hydrolysis probes were used in this reaction: a HEX-labelled probe specific for the intact proviral sequence and an unlabelled probe specific for the APOBEC-3G hypermutation, which competes with the HEX-labelled probe and results in no signal in the case of APOBEC hypermutations. Duplicate wells for IPDA were merged during analysis. All primer and probe details can be viewed in Supplementary Table 3.

### Targeted proteomic profiling

Plasma levels of CCL20 were measured using Human Quantikine ELISA kits (R&D Systems) according to the manufacturers protocol. Data for plasma CCL2 was used from our earlier publication^18^. In brief, plasma levels were analysed using proximity extension assay with the OLINK® immune-oncology panel (Olink, Sweden). Proteins are reported as normalised protein expression levels (NPX) where Ct values are normalised by the subtraction of the extension control and inter-plate control.

### Mass spectrometry-based proteomics

Proteins were extracted by adding 8 M urea in 100 mM ammonium bicarbonate lysis buffer. Samples were vortexed and sonicated in cold bath for 10 min. Protein concentration was estimated with Pierce Micro BCA assay (ThermoFisher Scientific). Extracted proteins were reduced with 10 mM dithiothreitol for 60 min at 37 °C and alkylated with 40 mM chloroacetamide (CAM) for 30 min at room temperature. Proteins were digested with trypsin (sequencing grade from Promega) with a 1:20 ratio enzyme:protein overnight at 37 °C. Resulting peptides were cleaned-up using C-18 stage tips (ThermoFisher Scientific) according to manufacturer’s instructions and dried in a vacuum concentrator (Eppendorf). Cleaned peptides were labelled with TMT11plex™ tags (ThermoFisher Scientific) following manufacturer’s instructions. Briefly, peptides were resuspended in 100 mM triethylammonium bicarbonate and individual TMT tags were dissolved in dry acetonitrile (ACN). Peptides were labelled by adding the respective TMT tag solution, the mix was incubated for 1 h at room temperature, the reaction was quenched by adding 5% hydroxylamine and incubated for 15 min at RT. Labelled peptides were mixed in three different batches according to the scheme described in Supplementary Table 4, a pool of samples was used across the three batches to normalize and evaluate batch effect (TMT channel 131C).

Labelled peptides were fractionated using Pierce High pH Reversed-Phase Peptide Fractionation Kit columns (ThermoFisher Scientific) following manufacturer’s instructions. Briefly, columns were washed with 100% ACN and then conditioned with 0.1% trifluoroacetic acid (TFA). Peptides were dissolved in 0.1% TFA and loaded into the column by centrifugation at 3000 × *g* for 2 min. Columns were washed with 5% ACN in 0.1% triethanolamine (TEA), and peptides were eluted in eight different fractions by centrifugation at 3000 × *g* according to the ACN concentration in 0.1% TEA solutions: 10, 12.5, 15, 17.5, 20, 22.5, 25 and 50%. All fractions were dried down in a vacuum concentrator (Eppendorf) prior to LC-MS/MS analysis.

Fractionated peptides were analysed on an Ultimate 3000 UHPLC (ThermoFisher Scientific) hyphenated to an Orbitrap™ Lumos™ mass spectrometer (ThermoFisher Scientific). Peptides were loaded in an Acclaim PepMap trap column, 2 μm × 75 μm ID × 2 cm (Thermo Scientific) and separated in an EASY-Spray™ HPLC Column, 2 μm × 75 μm ID × 500 mm (Thermo Scientific) using a 120 min linear gradient. Data were acquired in data-dependent acquisition (DDA) mode, isolating the top 20 most intense precursors at 120,000 mass resolution in the mass range of *m/z* 375–1400, maximum injection time (IT) of 50 ms and dynamic exclusion of 30 s, precursors were isolated with 0.7 Th width. MS/MS scans were obtained using high collision energy of 34%, resolution of 45,000 and maximum IT of 86 ms.

Proteins were searched against both SwissProt human and HIV-1 databases using the search engine Mascot Server v2.5.1 (MatrixScience Ltd) in Proteome Discoverer v2.5 (ThermoFisher Scientific) software environment allowing maximum two missed cleavages. Oxidation of methionine, deamidation of asparagine and glutamine, TMT6plex modification of lysine and N-termini were set as variable modifications, while carbamidomethylation of cysteine was used as fixed modification. The false discovery rate (FDR) was set to 1%.

### Bioinformatics analysis

The data were normalized using quantile method from the R/Bioconductor package NormalyzerDE v1.4.0. Missing data were imputed by employing the nearest-neighbour averaging method using impute.knn function from R package impute v1.60.0. Default setting was used for the data imputation. Technical variations in the data due to batch effect were removed using R function Combat from the package sva v3.34.0. Differential expressions analysis was performed with R/Bioconductor package Limma v3.42.2. Gene-set enrichment analysis was performed using R/Bioconductor package piano v2.2.0. Gene-level t-statistics given by Limma, and hallmark gene set downloaded from MSigDB were used to find significantly enriched gene-sets. Gene-sets with Benjamini–Hochberg adjusted *p*-values <0.2 were considered as significantly enriched. Volcano and bubble plots were created using R package ggplot2 v3.3.2.

### Statistics and reproducibility

Statistical analysis was performed using two-tailed Mann–Whitney U-test, and Wilcoxon ranked sum test or paired *t*-test for longitudinal data, based on the distribution of the data in Prism v8 (GraphPad Prism Software). Statistical significance was *p* < 0.05 for all analysis. Data were visualized using the package ggplot2 v.3.3.2 in Rstudio (v.1.3.1056). Correlation analysis was performed using Spearman’s correlation (*p* < 0.05) in Rstudio (v.1.3.1056). All assays were performed in technical duplicates except for the flow cytometry evaluation that was performed in one replicate per sample.

### Reporting summary

Further information on research design is available in the Nature Research Reporting Summary linked to this article.

## Supplementary information

Peer Review File

Supplementary Information

Description of Additional Supplementary Files

Supplementary Data 1

Reporting Summary

## Data Availability

The mass spectrometry proteomics data have been deposited to the ProteomeXchange Consortium via the PRIDE partner repository with the dataset identifier PXD027749. Raw data for FACS and flow cytometry are available from the corresponding authors upon request. The source data underlying most graphs used in this manuscript are provided as a Supplementary data file (Supplementary Data 1, excel).
