# Supplementary material for: Peripheral blood CD4+CCR6+ compartment differentiates HIV-1 infected or seropositive elite controllers from long-term successfully treated individuals
Source: Commun Biol. 2022 Apr 13;5:357. doi: 10.1038/s42003-022-03315-x (PMC9008025; doi:10.1038/s42003-022-03315-x)
Supplement: Supplementary file 2 — Supplementary Information [file 42003_2022_3315_MOESM2_ESM.pdf]

Supplementary Information

Peripheral blood CD4$^+$CCR6$^+$ compartment differentiates HIV-1 infected or seropositive elite controllers from long-term successfully treated individuals

Sara Svensson Akusjärvi[1,*], Shuba Krishnan[1], Bianca B. Jütte[2], Anoop T. Ambikan[1], Soham Gupta[1], Jimmy Esneider Rodriguez[3], Ákos Végvári[3], Maike Sperk[1], Piotr Nowak[4], Jan Vesterbacka[4], J. Peter Svensson[2], Anders Sönnerborg[1,4], and Ujjwal Neogi[1,5,6,*]

Affiliations

[1] Division of Clinical Microbiology, Department of Laboratory Medicine, Karolinska Institutet, ANA Futura, Campus Flemingsberg, 141 52 Stockholm, Sweden
[2] Department of Biosciences and Nutrition, Karolinska Institutet, Neo, Campus Flemingsberg, 141 83 Stockholm, Sweden
[3] Division of Chemistry I, Department of Medical Biochemistry and Biophysics, Karolinska Institutet, Campus Solna, 171 65 Stockholm, Sweden
[4] Division of Infectious Disease, Department of Medicine Huddinge, Karolinska Institutet, I73, Karolinska University Hospital, 141 86 Stockholm, Sweden
[5] Christopher S. Bond Life Sciences Centre, University of Missouri, Columbia, MO, 65211, USA
[6] Manipal Institute of Virology (MIV), Manipal Academy of Higher Education, Manipal, Karnataka, India

Supplementary Table 1: Treatment regimen

**Treatment regimen, *N* (%)**	**ART**
3TC/DTG	1 (1.8)
ABC/3TC/DRV/ritonavir	3 (5.5)
ABC/3TC/DTG	20 (37)
ABC/3TC/EFV	5 (9.3)
ABC/3TC/NVP	2 (3.7)
ABC/3TC/RPV	4 (7.4)
CAB/RPV*	1 (1.8)
DRV/COB/DTG	1 (1.8)
RAL/3TC/EFV	1 (1.8)
TAF/FTC/DTG	5 (9.3)
TAF/FTC/EFV	1 (1.8)
TDF/FTC/DTG	2 (3.7)
TDF/FTC/EFV	3 (5.5)
TDF/TFC/EVG/Cob	1 (1.8)
TDF/FTC/RPV	4 (7.4)

*3TC, Lamivudine; ABC, Abacavir; *CAB/RPV, Cabotegravir given together with rilpivirine intramusculary as long-acting drugs; COB,Cobicistat; DRV, Darunavir; r, ritonavir (boosterdose); DTG, Dolutegravir; EFV, Efavirenz; EVG,Elvitegravir; FTC, Emtricitabine; N, Number; NVP, Nevirapine; RAL, Raltegravir; RPV, Rilpivirine; TDF, Tenofovir Disoproxil; TAF, Tenofovir Alafenamide*

Supplementary Table 2: Patient characteristics of the longitudinal data

Parameter	T1	T2	P-values
N	10	10	NA
Gender, Female, *N* (%)	2 (20)	2 (20)	NA
**At sampling**			
Age in years, mean (SD)	52 (7.93)	55 (7.93)	0.4087#
CD4 count (cells/µL); median (IQR)	510 (460-557.5)	510 (370-700)	0.9847*
CD8 count (cells/µL); median (IQR)	500 (425-640)	430 (345-655)	0.7039*
CD4:CD8 ratio; median (IQR)	1.084 (0.7811-1.31)	1.214 (0.8712-1.479)	0.5490*
Years on treatment; median (IQR)	19 (16.75-23.25)	22 (19.75-26.25)	0.1152*
Viral Load; Log_{10} copies/mL (IQR)	0 (0-0)	0 (0-0)	0.4737*

IQR, Interquartile Range; N, Number; NA, Not Applicable; SD, Standard Deviation; **Mann-Whitney U-test; #Unpaired t-test*

Supplementary Table 3: Primers and probes used for quantification of HIV-1 DNA

**Total HIV DNA quantification**	
Beta globin probe	HEX-ATCCACGTTCACCTTGCCCCACA-TAM
Beta globin forward	AGGGCCTCACCACCAACTT
Beta globin reverse	GCACCTGACTCCTGAGGAGAA
HIV (HXB2) probe	FAM-AAGTAGTGTGTGCCCGTCTG-MGBEQ
HIV (HXB2) forward	GCCTCAATAAAGCTTGCCTTGA
HIV (HXB2) reverse	GGCGCCACTGCTAGAGATTTT
**FAM coupled probes + primers**	
RPP30 3' (ctrl) probe	/56-FAM/AGAGAGCAA/ZEN/CTTCTTCAAGGGCCC/3IABkFQ/
RPP30 3' forward	GTGTGAGTCAATCACTAGACAGAA
RPP30 3' reverse	AAACTGCAACAACATCATAGAGC
2-LTRcircle probe	/56-FAM/ACACTACTT/ZEN/GAAGCACTCAAGGCAAGCTTT/3IABkFQ/
2-LTRcircle forward	AACTAGGGAACCCACTGCTTAAG
2-LTRcircle reverse	TCCACAGATCAAGGATATCTTGTC
Pol probe	/56-FAM/AAGCCAGGA/ZEN/ATGGATGGCC/3IABkFQ/
Pol forward	GCACTTTAAATTTTCCCATTAGTCCTA
Pol reverse	CAAATTTCTACTAATGCTTTTATTTTTTC
Psi (Ψ) probe	/56-FAM/TTTTGGCGT/ZEN/ACTCACCAGT/3IABkFQ/
Psi (Ψ) forward	CAGGACTCGGCTTGCTGAAG
Psi (Ψ) reverse	GCACCCATCTCTCTCCTTCTAGC
**HEX coupled probes + primers**	
RPP30 (ctrl) probe	/5HEX/CTGACCTGA/ZEN/AGGCTCT/3IABkFQ/
RPP30 forward	GATTTGGACCTGCGAGCG
RPP30 reverse	GCGGCTGTCTCCACAAGT
5'LTR probe	/5HEX/AAGTAGTGT/ZEN/GTGCCCGTCTG/3IABkFQ/
5'LTR forward	GCCTCAATAAAGCTTGCCTTGA
5'LTR reverse	GGCGCCACTGCTAGAGATTTT
Env7781 probe	/5HEX/CCTTGGGTT/ZEN/CTTGGGA/3IABkFQ/
Env7781 forward	AGTGGTGCAGAGAGAAAAAAGAGC
Env7781 reverse	GTCTGGCCTGTACCGTCAGC
**Uncoupled probes**	
Env 7781 hypermutation probe	CCTTAGGTTCTTAGGAGC/3IABkFQ/

Supplementary Table 4: TMT11plex labelling distribution for CCR6$^+$ and CCR6$^-$ samples

B1		B2		B3	
ART01 +	126	ART03 +	127N	ART05 +	131N
ART01 -	127N	ART03 -	128C	ART05 -	130N
ART02 +	127C	ART04 +	129N	ART06 +	129N
ART02 -	128N	ART04 -	130C	ART06 -	128N
EC01 +	128C	EC03 +	131N	EC05 +	127N
EC01 -	129N	EC03 -	130N	EC05 -	126
EC02 +	129C	EC04 +	129C	EC06 +	130C
EC02 -	130N	EC04 -	128N	EC06 -	127C
HC02 +	130C	HC01 +	127C	HC03 +	128C
HC02 -	131N	HC01 -	126	HC03 -	129C
Norm	131C	Norm	131C	Norm	131C

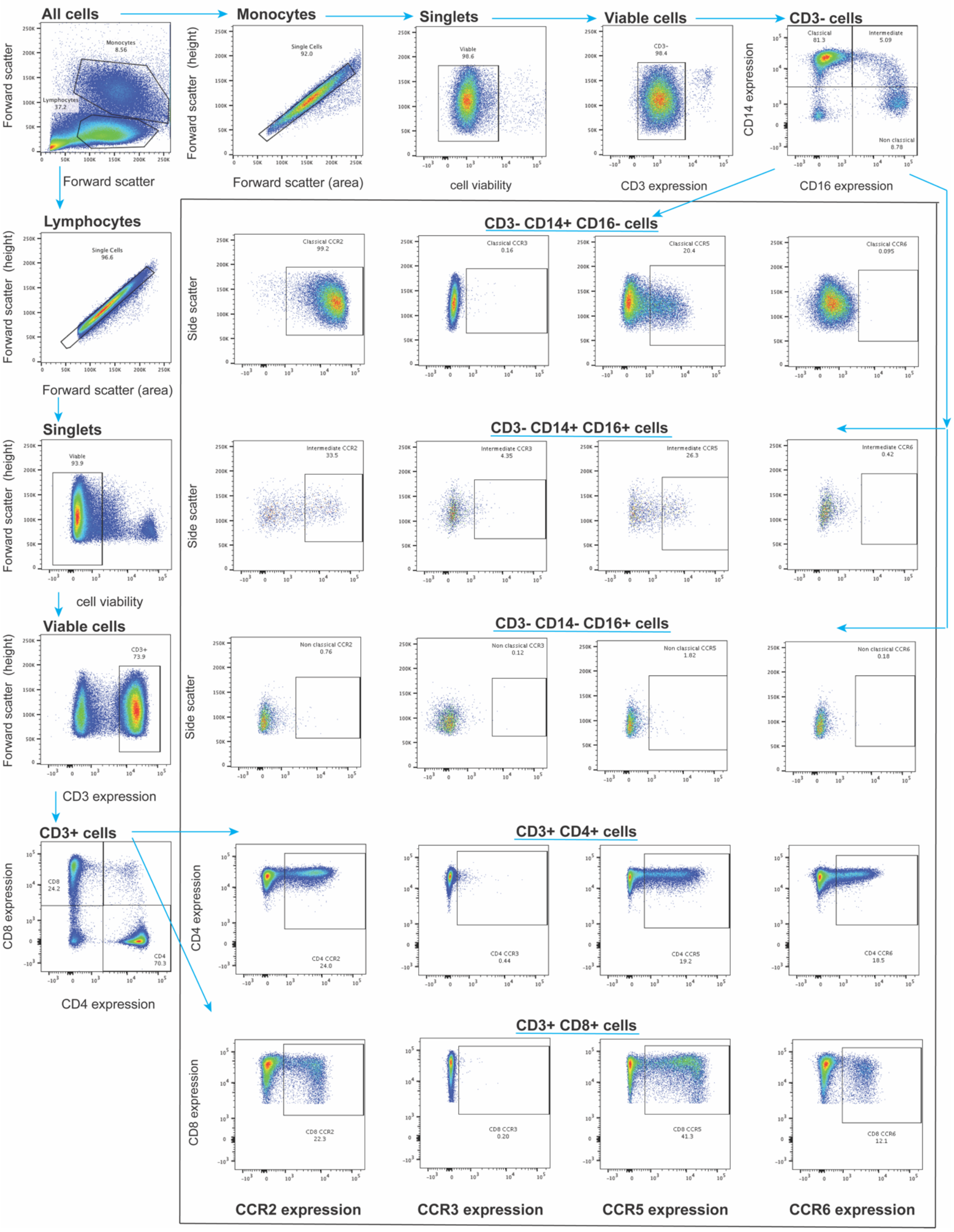

Supplementary Fig. 1: Gating strategy for CCR2, CCR3, CCR5, and CCR6 flow cytometry detection

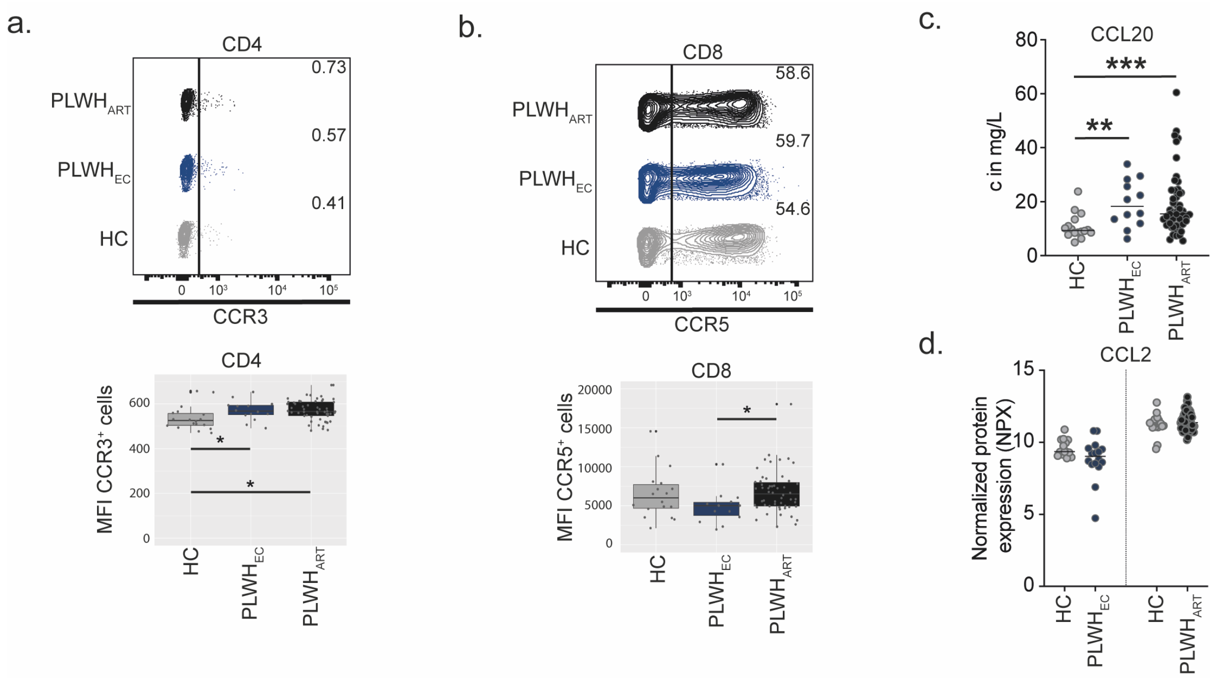

Supplementary Fig. 2: Chemokine receptor expression on lymphocytic cell populations.
Flow cytometry detection in lymphocytic cell populations in $PLWH_{EC}$ (*n=14*), $PLWH_{ART}$ (*n=54*), and HC (*n=18*). **a** Expression of CCR3 in $CD4^+$ T cells. The contour plot is a median representative sample of % of cells expressing CCR3, and the boxplot shows the median fluorescence intensity (MFI). **b** Expression of CCR5 in $CD8^+$ T cells. The contour plot is a median representative sample of % of cells expressing CCR5, and the boxplot shows the median MFI. **c** Chemokine detection of CCL20 in plasma of $PLWH_{EC}$ (*n=12*), $PLWH_{ART}$ (*n=49*), and HC (n=*16*). **d** Normalized expression profile of CCL2 plasma levels in $PLWH_{EC}$ (*n=14*), $PLWH_{ART}$ (*n=51*), and HC (n=*17*). Chemokine detection by ELISA was performed in technical duplicates. Statistical significance was determined using two-tailed Mann-Whitney U-test (significance level $p<0.05$, with $* < 0.05$, $** < 0.001$) and represented with median and 95% CI.

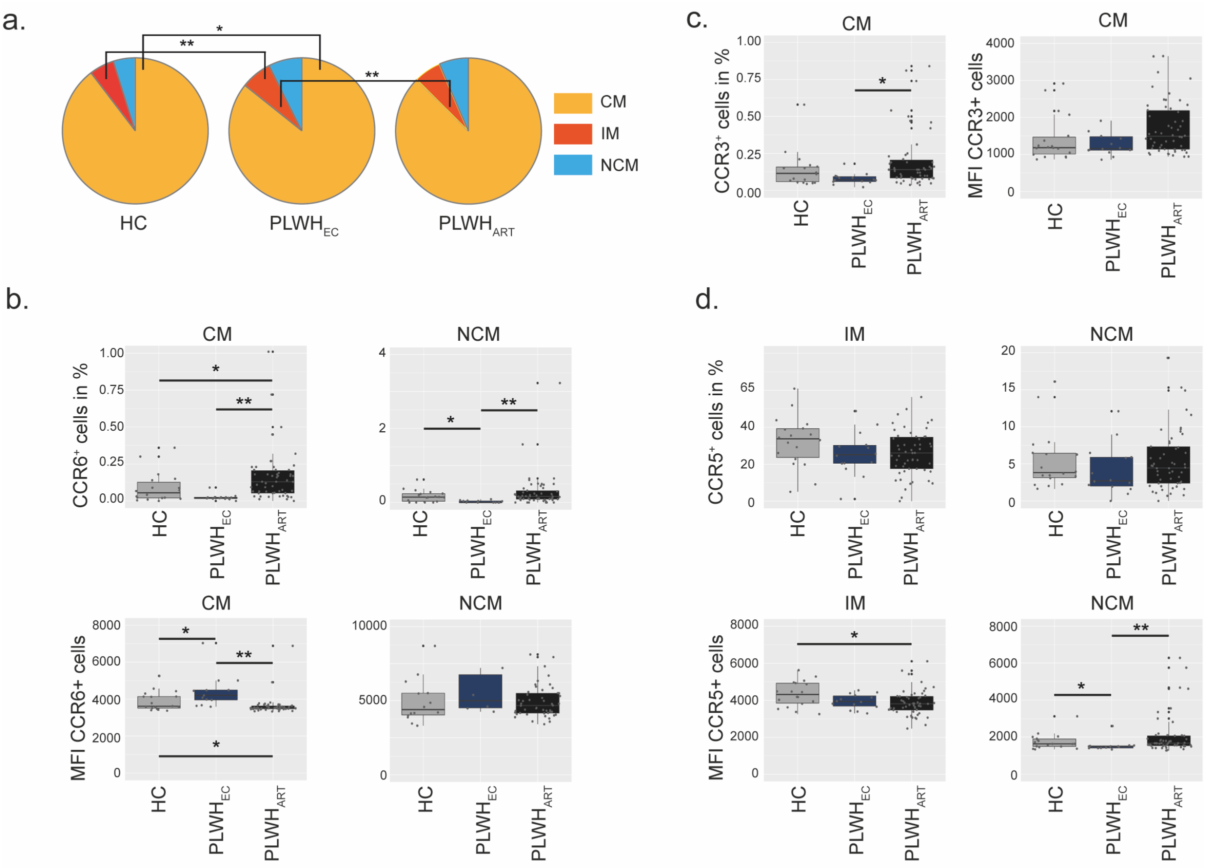

Supplementary Fig. 3: Chemokine receptor expression on monocytic cell population.
Flow cytometry detection in monocytic cell populations (classical monocytes (CM); $CD14^{+}CD16^{-}$, intermediate monocytes (IM); $CD14^{+}CD16^{+}$, and non-classical monocytes (NCM); $CD14^{-}CD16^{+}$) in $PLWH_{EC}$ (*n=14*), $PLWH_{ART}$ (*n=54*), and HC (*n=18*). **a** Frequency distribution of CM, IM, and NCM. **b** Expression of CCR6 in CM and NCM cells. The upper row shows % of cells expressing the receptor and the bottom row shows the median fluorescence intensity (MFI). **c** Expression of CCR3 in CM. The left boxplot shows % of cells expressing the receptor and the right boxplot shows the MFI. **d** Expression of CCR5 in IM and NCM. The upper row shows % of cells expressing the receptor and the bottom row shows the MFI. Statistical significance was determined using two-tailed Mann-Whitney U-test (significance level $p<0.05$, with $* < 0.05$, $** < 0.001$) and represented as pie charts or with median using 95% CI.

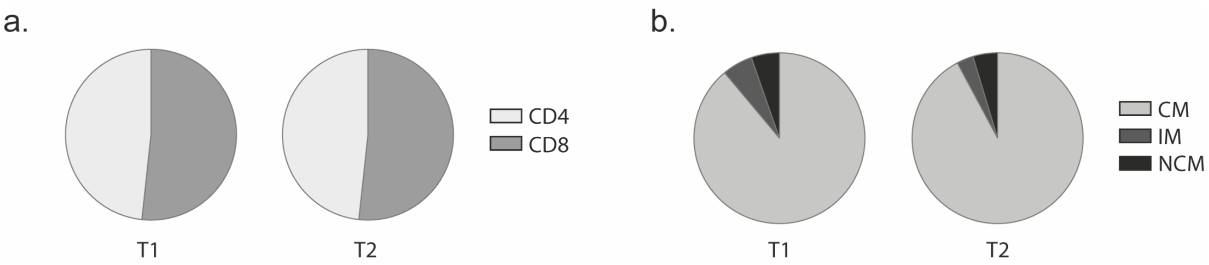

Supplementary Fig. 4: Frequency of cell populations in longitudinal data of $PLWH_{ART}$.
Flow cytometry analysis of longitudinal dataset of HIV-1 infected individuals (timepoint 1 (T1), *n=10* and timepoint 2 (T2), *n=10*). **a, b** Frequency of lymphocytic cell populations ($CD4^+$ and $CD8^+$) (**a**) and monocytic cell populations (classical monocytes (CM); $CD14^+CD16^-$, intermediate monocytes (IM); $CD14^+CD16^+$, and non-classical monocytes (NCM); $CD14^-CD16^+$) (**b**) in T1 and T2.

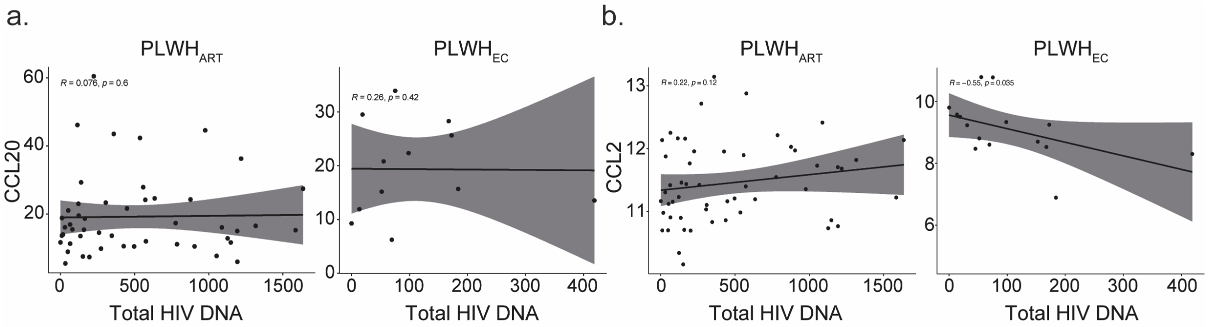

Supplementary Fig. 5: Correlation of chemokines CCL20 and CCL2 to total HIV-1 DNA.
a, **b** Correlation between total HIV-1 DNA and plasma levels of CCL20 (**a**) and CCL2 (**b**) in $PLWH_{ART}$ and $PLWH_{EC}$. Statistical significance was tested using Spearman's correlation.

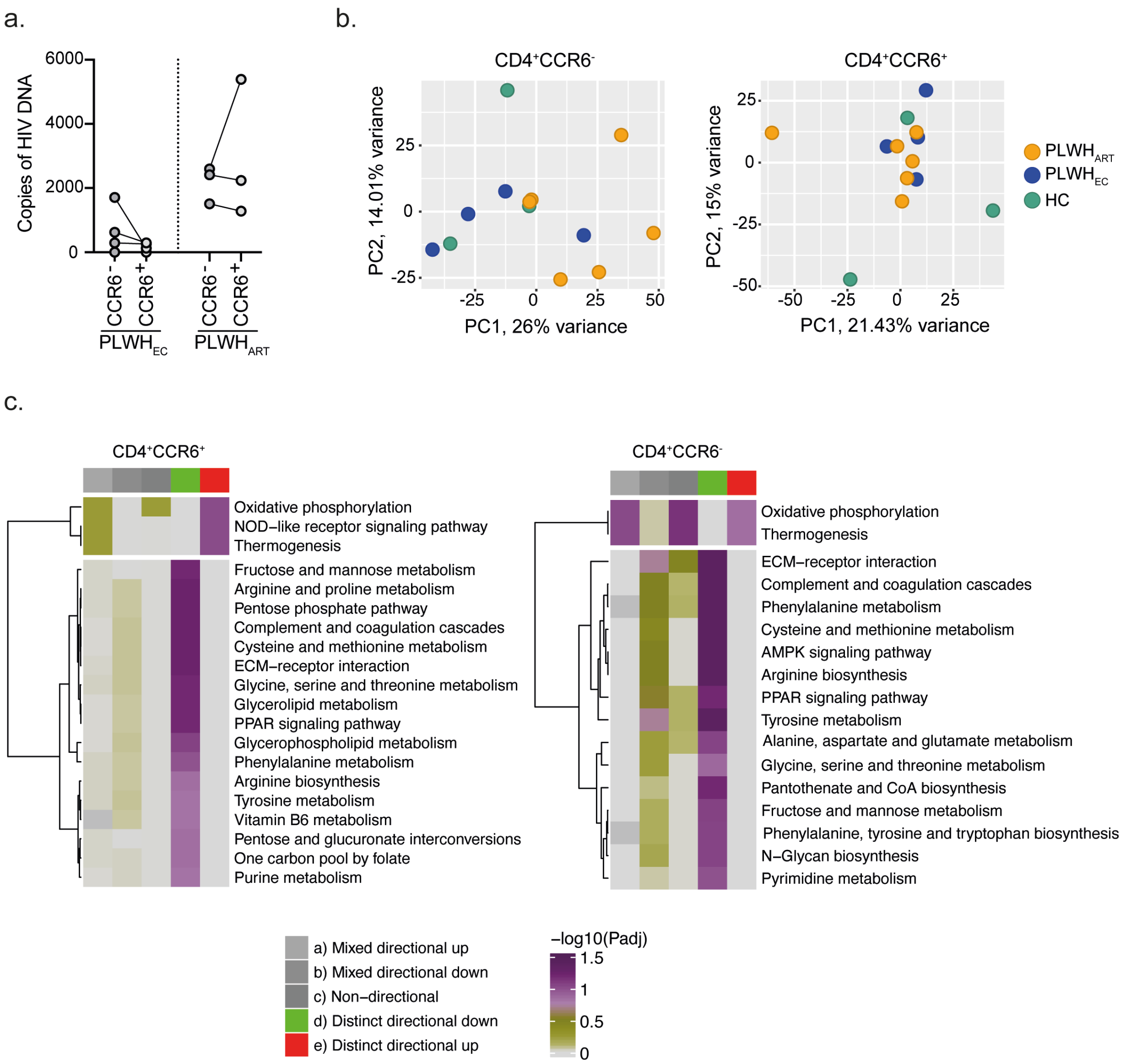

Supplementary Fig. 6: Analysis of CD4$^+$CCR6$^+$ and CD4$^+$CCR6$^-$ cell populations in PLWH$_{EC}$ compared to PLWH$_{ART}$.

a HIV-1 DNA quantified in CD4$^+$CCR6$^+$ and CD4$^+$CCR6$^-$ cells from PLWH$_{EC}$ (*n=5*) and PLWH$_{ART}$ (*n=3*). Data is represented as paired datapoints where line is drawn within between the two populations in the same patient. The quantification was performed in technical duplicates. **b** Two-dimensional visualization of PC1 and PC2 of principal components analysis using CD4$^+$CCR6$^-$ and CD4$^+$CCR6$^+$ samples. **c** Heatmap representation of significant pathways found differentially regulated in CD4$^+$CCR6$^+$ PLWH$_{EC}$ compared to CD4$^+$CCR6$^+$ PLWH$_{ART}$ and in CD4$^+$CCR6$^-$ PLWH$_{EC}$ compared to CD4$^+$CCR6$^-$ PLWH$_{ART}$ using KEGG 2021. Color gradient is corresponding to the negative log scaled adjusted p-values. Each column represents p-values of various directionality classes, calculated for the pathways. Non-directional p-values are calculated based on gene-level statistics regardless of the direction of expression. Mixed directional up and mixed directional down p-values are calculated using the subset of the gene statistics that are up-regulated and down-regulated, respectively. Distinct directional up and distinct directional down p-values are calculated from gene statistics with expression direction.

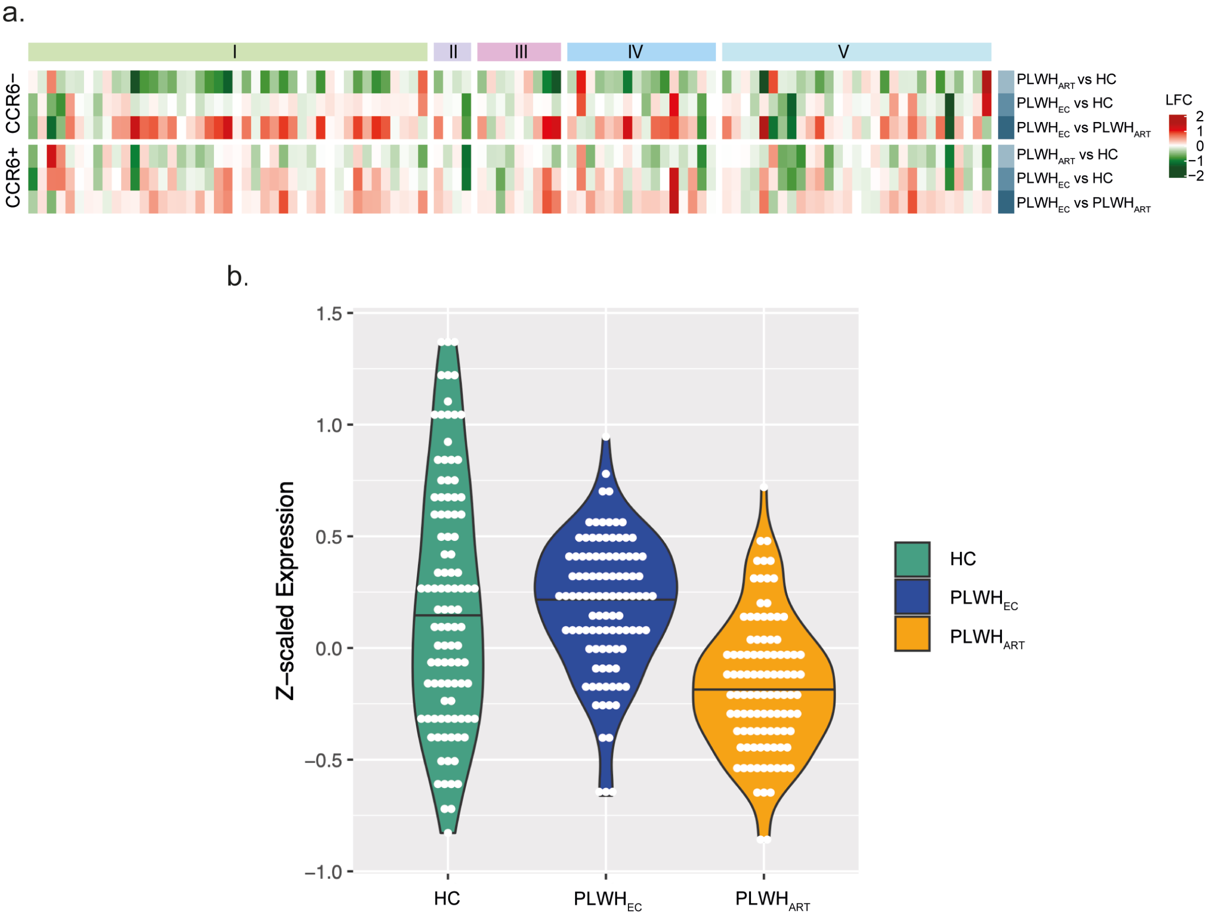

Supplementary Fig. 7: Abundance of OXPHOS proteins detected from the proteomics analysis of $CD4^{+}CCR6^{+}$ and $CD4^{+}CCR6^{-}$ cells.

a Heatmap visualization of regulation of OXPHOS pathway proteins detected in the proteomics experiment. Color gradient represents log2 scaled fold change values. Column annotation denotes each of the five complexes of OXPHOS pathway. Row annotation denotes each of the pair-wise analysis performed. **b** Cumulative Z-scale expression of the OXPHOS genes.
