# Supplementary material for: Peripheral blood CD4+CCR6+ compartment differentiates HIV-1 infected or seropositive elite controllers from long-term successfully treated individuals
Source: Commun Biol. 2022 Apr 13;5:357. doi: 10.1038/s42003-022-03315-x (PMC9008025; doi:10.1038/s42003-022-03315-x)
Supplement: Supplementary file 3 — Description of Additional Supplementary Files [file 42003_2022_3315_MOESM3_ESM.pdf]

Description of Additional Supplementary Files

File name: Supplementary Data 1
Description: The source data underlying most graphs used in this manuscript.
