# Supplementary material for: Peripheral blood CD4+CCR6+ compartment differentiates HIV-1 infected or seropositive elite controllers from long-term successfully treated individuals
Source: Commun Biol. 2022 Apr 13;5:357. doi: 10.1038/s42003-022-03315-x (PMC9008025; doi:10.1038/s42003-022-03315-x)
Supplement: Supplementary file 5 — Reporting Summary [file 42003_2022_3315_MOESM5_ESM.pdf]

nature research

Corresponding author(s): Sara Svensson Akusjärvi
Ujjwal Neogi

Last updated by author(s): Mar 17, 2022

Reporting Summary

Nature Research wishes to improve the reproducibility of the work that we publish. This form provides structure for consistency and transparency in reporting. For further information on Nature Research policies, see our Editorial Policies and the Editorial Policy Checklist.

Statistics

For all statistical analyses, confirm that the following items are present in the figure legend, table legend, main text, or Methods section.

n/a	Confirmed	
☐	☒	The exact sample size (*n*) for each experimental group/condition, given as a discrete number and unit of measurement
☐	☒	A statement on whether measurements were taken from distinct samples or whether the same sample was measured repeatedly
☐	☒	The statistical test(s) used AND whether they are one- or two-sided<br>*Only common tests should be described solely by name; describe more complex techniques in the Methods section.*
☒	☐	A description of all covariates tested
☐	☒	A description of any assumptions or corrections, such as tests of normality and adjustment for multiple comparisons
☐	☒	A full description of the statistical parameters including central tendency (e.g. means) or other basic estimates (e.g. regression coefficient) AND variation (e.g. standard deviation) or associated estimates of uncertainty (e.g. confidence intervals)
☐	☒	For null hypothesis testing, the test statistic (e.g. *F*, *t*, *r*) with confidence intervals, effect sizes, degrees of freedom and *P* value noted<br>*Give P values as exact values whenever suitable.*
☒	☐	For Bayesian analysis, information on the choice of priors and Markov chain Monte Carlo settings
☒	☐	For hierarchical and complex designs, identification of the appropriate level for tests and full reporting of outcomes
☐	☒	Estimates of effect sizes (e.g. Cohen's *d*, Pearson's *r*), indicating how they were calculated

Our web collection on statistics for biologists contains articles on many of the points above.

Software and code

Policy information about availability of computer code

Data collection	Proteomics was performed using TMT LS-MS/MS. Proteins were searched against both SwissProt human and HIV-1 databases using the search engine Mascot Server v2.5.1 (MatrixScience Ltd) in Proteome Discoverer v2.5 (ThermoFisher Scientific) software environment.
Data analysis	Analysis of proteomics data was performed using a custom code which will be available in the manuscript before publication (Link: https://github.com/neogilab/CCR6). In brief, R/Bioconductor package NormlayzerDE v1.4.0 was used, impute.knn function v1.60.0. Technical variations removed using Combat from the package sva v3.34.0 and differential expression analysis using R/bioconductor package Limma v3.42.2. Gene set enrichment analysis was performed using the package piano v2.2.0. Gene level t-stagtistics givven by Limma and hallmark gene set downloaded from MSigDB were used to find significantly enriched gene-sets. Benjamini-Hochberg adjusted p-value less than 0.2 were considered as signifcantly enriched. Data was represented using Volcano and bubble plots with ggplot2 v3.3.2.<br>Other statistics was performed using two-tailed Mann-Whiteny U test, Wilcoxon ranked sum test or paired t-test for longitudinal data in Prism v8 (GraphPad Prism Software). Spearman's correlation was performed in Rstudio (v.1.3.1056).

For manuscripts utilizing custom algorithms or software that are central to the research but not yet described in published literature, software must be made available to editors and reviewers. We strongly encourage code deposition in a community repository (e.g. GitHub). See the Nature Research guidelines for submitting code & software for further information.

Data

Policy information about availability of data

All manuscripts must include a data availability statement. This statement should provide the following information, where applicable:

- Accession codes, unique identifiers, or web links for publicly available datasets
- A list of figures that have associated raw data
- A description of any restrictions on data availability

The mass spectrometry proteomics data have been deposited to the ProteomeXchange Consortium via the PRIDE partner repository with the dataset identifier PXD027749.

Field-specific reporting

Please select the one below that is the best fit for your research. If you are not sure, read the appropriate sections before making your selection.

☒ Life sciences ☐ Behavioural & social sciences ☐ Ecological, evolutionary & environmental sciences

For a reference copy of the document with all sections, see nature.com/documents/nr-reporting-summary-flat.pdf

Life sciences study design

All studies must disclose on these points even when the disclosure is negative.

Sample size	No specific sample size were calculated. Feasibility sampling. Blood samples were collected.This study included three groups of individuals from the Swedish InfCare Cohort. This included HIV-1 negative controls (HC, n=18), people living with HIV-1 on antiretroviral therapy (PLWHART, n=54), and people living with HIV-1 with natural control of infection (PLWHEC, n=14)
Data exclusions	none
Replication	Experiments was performed in techical replicats when applicable. Does not include Flow cytometry
Randomization	na
Blinding	none

Reporting for specific materials, systems and methods

We require information from authors about some types of materials, experimental systems and methods used in many studies. Here, indicate whether each material, system or method listed is relevant to your study. If you are not sure if a list item applies to your research, read the appropriate section before selecting a response.

Materials & experimental systems

n/a	Involved in the study	
☐	☒	Antibodies
☒	☐	Eukaryotic cell lines
☒	☐	Palaeontology and archaeology
☒	☐	Animals and other organisms
☐	☒	Human research participants
☐	☒	Clinical data
☒	☐	Dual use research of concern

Methods

n/a	Involved in the study	
☒	☐	ChIP-seq
☐	☒	Flow cytometry
☒	☐	MRI-based neuroimaging

Antibodies

Antibodies used	CD3 (OKT3, FITC, Biolegend #317306), CD4 (SK3, BUV395, BDbioscience #563550), CD8 (RPA-T8, APC, Biolegend #301014), CD14 (M5E2, BV510, Biolegend #301842), CD16 (3G8, BV786, BDbioscience #563690), CCR2 (1D9, BB700, BDbioscience #747847), CCR3 (5E8, BV421, Biolegend #310714), CCR5 (2D7, PE-CF594, Bdbioscience #562456), CCR6 (G034E3, BV711, Biolegend #353436), CD4 (SK3, PE-Cy5, Biolegend # 344654), CD8 (RPA-T8, BV570, Biolegend #301038)
Validation	commersial

Human research participants

Policy information about studies involving human research participants

Population characteristics	age, gender, clinical parameters like (CD4, CD8, viral load), treatment duration, treatment regimen
Recruitment	na
Ethics oversight	The study was approved by Etikprövningsmyndigheten (Sweden) with ethical clearance Dnr 2013/1944-31/4 and amendment 2019-05585 together with ethical clearance Dnr 2009/1485-31 and amendment 2019-05584. Informed consent was obtained from all study participants before inclusion and kept at the respective sites.

Note that full information on the approval of the study protocol must also be provided in the manuscript.

Clinical data

Policy information about clinical studies

All manuscripts should comply with the ICMJE guidelines for publication of clinical research and a completed CONSORT checklist must be included with all submissions.

Clinical trial registration	na
Study protocol	na
Data collection	na
Outcomes	na

Flow Cytometry

Plots

Confirm that:

☒ The axis labels state the marker and fluorochrome used (e.g. CD4-FITC).

☒ The axis scales are clearly visible. Include numbers along axes only for bottom left plot of group (a 'group' is an analysis of identical markers).

☒ All plots are contour plots with outliers or pseudocolor plots.

☒ A numerical value for number of cells or percentage (with statistics) is provided.

Methodology

Sample preparation	As per manufacturers instruction
Instrument	Symphony flow cytometer (BD Bioscience) or SONY MA900 (SONY cooperation)
Software	FlowJo 10.6 (TreeStar Inc)
Cell population abundance	The original abundance of the CD4+CCR6+ and CD4+CCR6- cell fractions were determined based on the first flow cytometry experiment to range between 4-27% for CCR6+ and 73-96% for CCR6- of total CD4+ T cells. Sort efficiency of the instrument ranged from 81-98%. The purity of sorted cell populations was assessed by running a small fraction of sorted cells on the cytometer again. From this the mean purity was 86.3% in the samples.
Gating strategy	Available in the supplementary or in corresponding figure

☒ Tick this box to confirm that a figure exemplifying the gating strategy is provided in the Supplementary Information.
